# Alignment of spatial genomics data using deep Gaussian processes

**DOI:** 10.1038/s41592-023-01972-2

**Published:** 2023-08-17

**Authors:** Andrew Jones, F. William Townes, Didong Li, Barbara E. Engelhardt

**Affiliations:** 1 Department of Computer Science, Princeton University, Princeton, NJ USA; 2 Department of Statistics and Data Science, Carnegie Mellon University, Pittsburgh, PA USA; 3 Department of Biostatistics, University of North Carolina, Chapel Hill, NC USA; 4 Gladstone Institutes, San Francisco, CA USA; 5 Department of Biomedical Data Science, Stanford University, Stanford, CA USA

**Keywords:** Machine learning, Statistical methods, Image processing, Software, Transcriptomics

## Abstract

Spatially resolved genomic technologies have allowed us to study the physical organization of cells and tissues, and promise an understanding of local interactions between cells. However, it remains difficult to precisely align spatial observations across slices, samples, scales, individuals and technologies. Here, we propose a probabilistic model that aligns spatially-resolved samples onto a known or unknown common coordinate system (CCS) with respect to phenotypic readouts (for example, gene expression). Our method, Gaussian Process Spatial Alignment (GPSA), consists of a two-layer Gaussian process: the first layer maps observed samples’ spatial locations onto a CCS, and the second layer maps from the CCS to the observed readouts. Our approach enables complex downstream spatially aware analyses that are impossible or inaccurate with unaligned data, including an analysis of variance, creation of a dense three-dimensional (3D) atlas from sparse two-dimensional (2D) slices or association tests across data modalities.

## Abstract (web summary)

Gaussian Process Spatial Alignment (GPSA) aligns multiple spatially resolved genomics and histology datasets and improves downstream analysis.

## Main

Spatially-resolved genomic technologies hold the promise to understand the spatial organization, variation and local effects of cellular morphology, gene expression, protein expression and other cellular phenotypes^1–10^. As new technologies have been developed, several computational models and analysis pipelines have been proposed for processing and downstream analyses of single-slice data^11–17^.

Although these technologies and methods have enabled scientific discoveries, it remains difficult to jointly analyze multiple phenotypic readouts from these technologies due to inevitable spatial warping and biological variation across slices, samples and individuals. Furthermore, the various spatial genomic platforms range widely in field of view, spatial resolution and number of phenotypic readouts that they measure. The standard analysis, in which each slice is analyzed separately, reduces the statistical power of the analyses or prohibits these analyses entirely. Thus, there remains a need for tools that enable a joint analysis across slices, samples, modalities and technologies.

The problem of integrating disparate spatially resolved samples arises in several fields. Spatial alignment has been well studied in the context of functional magnetic resonance imaging (fMRI) brain data^18–20^. At a given time point, an fMRI scan produces measurements on a 3D grid across the brain across time, where the continuous level of blood flow is measured at each point (‘voxel’) in the grid. Given multiple scans across days or individuals, the alignment problem is to warp the spatial coordinates of each voxel in a scan so that the (*x*, *y*, *z*) voxels in each patient refer to approximately the same functional voxel in the brain.

Two major types of fMRI alignment have emerged: template-based registration and hyperalignment. Template-based registration methods seek to align scans from different individuals to a pre-defined CCS. This CCS is typically defined as a single individual’s scan or as the average across multiple manually aligned scans. The most popular approach uses a ‘template brain’ developed at the Montreal Neurological Institute^21,22^. Next, fMRI samples’ voxel coordinates are warped such that the new samples’ voxel coordinates match this template in terms of both relative location in the brain and voxel behavior across time. Hyperalignment approaches seek to align different individuals’ data without a pre-defined template. In particular, hyperalignment methods compute scan-specific transformations of voxel space using the centroid of all scans as the CCS. Both linear^23^ and nonlinear^24^ hyperalignment approaches have been developed.

Alignment methods for fMRI data are not easily extensible to spatial genomic and histology images for three reasons. First, curated anatomical CCSs are not available for the diversity of tissue types, developmental stages and species that are studied using spatial genomics. Second, while the readout at each location for fMRI data is a single number representing blood flow, the readout in spatial genomics often has 10^2^–10^5^ sparse features. Finally, while the spatial resolution of fMRI scans tends to be one of a few standard resolutions, there is a wide diversity of spatial technologies, each with their own resolution and field of view. Thus, there is a need for spatial genomic-specific alignment methods.

In spatial genomics, we are aware of four approaches for aligning samples’ spatial coordinates. Probabilistic alignment of spatial transcriptomic (ST) experiments (PASTE)^25^ was developed to align adjacent tissue slices in ST data^1^. PASTE uses an optimal transport framework to identify mappings between the spatial locations of adjacent slices. Its objective function trades off transcriptional similarity and proximity of spatial locations. While PASTE is robust and fast, it is limited to linear alignments, which are often insufficiently expressive for complex distortions of data. While alignment is not the focus of Splotch^26^, the method uses a linear autocorrelation model to shift ST slices to align specific tissue regions.

Two landmark-based approaches to spatial alignment have also been proposed. Effortless generic Gaussian process (GP) landmark transfer (Eggplant) was developed using GP regression^27^. Eggplant projects gene expression values of each misaligned slice onto a given CCS. Eggplant requires the user to identify a set of landmarks on each misaligned sample and the template sample. Eggplant performs this template transfer independently for each slice and each gene, ignoring any correlation between them. ST imaging framework (STIM) borrows techniques from computer vision to register ST data into a CCS^28^. For both Eggplant and STIM, the identification of shared landmark locations may be difficult across slices from tissues without canonical structures, such as tumors.

In this work, we present a probabilistic model that aligns the coordinates of spatial genomic samples across tissue slices, individuals and data modalities. We apply our model to ST data and, in one experiment, paired histology data, at subcellular, cellular and supercellular resolutions from different platforms. Given a set of unaligned slices, our approach iteratively estimates a robust CCS (shared spatial locations capturing the full breadth of the slices) and maps the local coordinates from each slice onto the CCS. Our model, which leverages similarities in both spatial structure and phenotypic readouts between slices, enables the creation of a CCS onto which heterogeneous slices may be mapped and then analyzed jointly with respect to the shared CCS. The automated creation of a CCS is itself a contribution; few CCSs exist because of the challenges in creating them^29,30^.

Our proposed generative model uses two stacked GPs to align spatial slices across samples and technologies in a 2D, 3D or potentially four-dimensional spatiotemporal coordinate system. Given a location in a slice, the first layer maps this location to the corresponding location in the CCS. The second layer generates the distribution of phenotypic readouts at that location (for example, the distribution of gene expression values). Together, the first layer representing a CCS and the second layer representing a map from each location in the CCS to estimates of phenotypic readouts represent an atlas. Our approach opens the door to de novo creation of large tissue atlases using collections of tissue samples. Our model allows for straightforward downstream analyses on the aligned slices, including imputation of sparse measurements, analysis of variation and joint mapping of slices with distinct modalities from different technologies.

## Results

### GPSA

Our GPSA is a Bayesian model for aligning spatial genomic and histology samples with spatial coordinates that are distorted or on different systems. Each slice is assigned its own warping function in the first layer of GPSA, which accounts for slice-specific deformations. In the second layer, GP functions model phenotypic readouts at each location in the CCS. Inference is guided by two competing objectives: retain the current position of each spot in a slice while warping each spot to ensure that the readouts within each warped slice match as closely as possible with the readout distributions encoded in the second layer of the deep GP (DGP). See Methods for details.

Although readouts are typically high dimensional, the readout features tend to be correlated, and this structure may be captured by a low-dimensional manifold^31–34^. Thus, GPSA models the readouts as a weighted linear combination of a small number of GPs through a linear model of coregionalization (LMC^35^; see Methods for details).

GPSA allows for joint modeling of multiple types of readout modalities. For example, many experiments collect both spatial expression profiles and histology images for each slice^1,36^. These modalities contain complementary information, and it is of interest to analyze both modalities across multiple slices jointly. To do this, we augment our model of the phenotypic readouts (Equation (1)) to include a separate likelihood for each modality, allowing for straightforward multimodal alignment. See Methods for details.

### De novo and template-based CCSs

We propose two methods for aligning slices using GPSA: de novo alignment and template-based alignment. A de novo alignment estimates a CCS from scratch using the slices while simultaneously projecting these slices onto the CCS. Alternatively, if a CCS exists for a tissue and context of interest, a template-based alignment maps the samples to this given CCS. This is accomplished by fixing the warping function of the CCS to the identity. In practice, to avoid extreme warps in de novo alignment, we recommend arbitrarily choosing one of the input samples to fix as the CCS.

### Simulations

We first validate the accuracy and robustness of our model using synthetic data generated under a variety of settings.

#### Recovery of true latent common coordinates

**Fig. 1:**
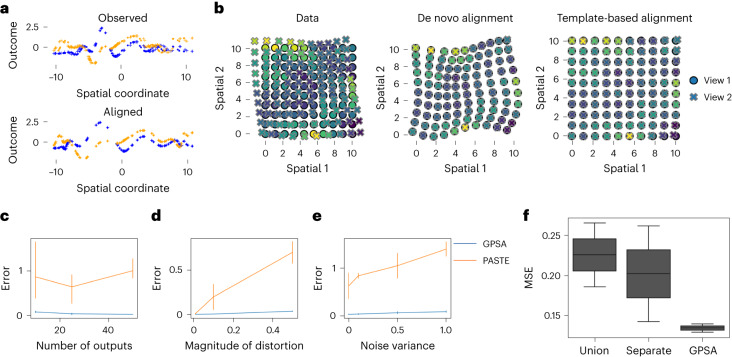
Demonstration of GPSA with synthetic data. **a**, GPSA applied to a 1D spatial coordinate system with *p* = 2 readout features (blue and orange) and *S* = 2 slices (dots and crosses). The *x* axis shows the spatial coordinate of each sample, and the *y* axis shows the readout values. Top, observed data; bottom, the CCS. **b**, GPSA applied to 2D data with *p* = 10 readout features and *S* = 2 slices (dots and crosses). The *x* and *y* axes show the spatial coordinates of each sample. Points are colored by one feature value. Left, observed data. Middle and right, de novo and template-based alignments from GPSA. **c**, Alignment error (MSE between all pairwise aligned slices with shared original coordinates) of GPSA and PASTE^25^ across varying numbers of readout features. Error bars capture the s.d. across five random runs. **d**, Similar to **c** but shows the error across varying levels of distortion within the slices. **e**, Similar to **c**,**d** but shows the error across different levels of variance in the synthetic expression data. **f**, Prediction with synthetic data. MSE for prediction of readout values versus ground truth on held-out spots from an aligned slice. ‘Union’ represents predictions from a GP fit to a concatenation of the observed samples, ‘separate’ refers to predictions from independent GPs fit to each sample separately, and ‘GPSA’ refers to predictions from GPSA fit across all samples. Each box plot shows the minimum and the maximum values (whiskers), the median (center line) and the first and third quartiles (the box boundary).

First, we generated synthetic spatial expression data for two slices from a known CCS, and we began with a one-dimensional CCS to study and visualize the behavior of GPSA. We sampled spatial coordinates for *n* = 100 locations in the interval (0, 10). We then generated observed spatial coordinates for *S* = 2 slices by applying a GP warp (see Methods for details). We sampled synthetic gene expression *y*_*ij*_ for gene *j* using a GP:

1 $${y}_{ij}=f({x}_{i}^{\star })+\epsilon ,\,\,f \sim {{{\rm{GP}}}}(0,k),\,\,\epsilon \sim {{{\mathcal{N}}}}(0,{\sigma }^{2}),$$

where ${x}_{i}^{\star }$ is the location of the *i*th sample, 𝜖 is the local Gaussian error, f is a random nonlinear function generated from a GP, and σ2 is the variance term for the Gaussian error. We set *k* to be the radial basis function (RBF) with hyperparameter *τ*^2^ = 0.1 (Methods). We fit GPSA to this dataset using a de novo alignment and extracted the aligned coordinates for each slice. We found that the warped coordinates were well aligned between the two slices and that the relationship between spatial coordinates and gene expression was well preserved (Fig. 1a). The mean squared error (MSE) for the aligned coordinates was 0.000134 (where an MSE of 0 indicates perfect performance), while the MSE of the original spatial coordinates was 0.0345. This result suggests that GPSA is able to align distorted and disparate samples accurately.

Next, we extended this experiment to a more realistic setting in which the spatial coordinates are 2D. Here, the CCS was a 15 × 15 grid containing *n* = 225 spatial locations. The observed spatial coordinates for the first slice were kept at the original grid, and the observed coordinates for the second slice were generated by randomly warping the CCS with a GP warp (Methods) using an isotropic RBF with length scale *ℓ* = 10 and spatial variance *τ*^2^ = 0.5. We sampled synthetic expression values from a GP with the true spatial coordinates as inputs (Equation 1). We fit the model twice with this dataset: once using a de novo alignment and once using a template-based alignment with the first slice as the template. For both alignments, the warped coordinates were aligned with minimal distortion using the latent CCS (Fig. 1b). The MSEs for the de novo and template-based aligned coordinates were 0.000537 and 0.00725, respectively, while the MSE of the original spatial coordinates was 0.733. These results indicate that GPSA is a viable model for aligning distorted spatially-resolved slices using both de novo and template-based alignment.

#### Robustness of GPSA to observation noise

We next tested GPSA’s robustness to the number of readout features, the magnitude of distortion between the slices relative to the CCS and the noise variance in the readouts. To do so, we generated synthetic datasets with 2D spatial coordinates using the same approach as before. We varied the number of readout features *p* ∈ {1, 20, 50}. To vary the magnitude of distortion between slices, we varied the spatial variance *τ*^2^ of the RBF kernel of the GP warp (Methods; Equation 10), which corresponds to a larger distortion between slices. We fit GPSA to each of these datasets using a template-based alignment with the first slice as the template, repeating the experiment five times for each condition.

For comparison, we ran PASTE^25^ and extracted the aligned coordinates. Every pair of simulated slices contains spots at identical locations. We measured the error between the warped locations for each of these spots between every pair of slices: $\frac{1}{Sn}{\sum }_{s < {s}^{{\prime} }}\mathop{\sum }\nolimits_{i = 1}^{n}\parallel {\widetilde{{{{\bf{x}}}}}}_{i}^{s}-{\widetilde{{{{\bf{x}}}}}}_{i}^{{s}^{{\prime} }}{\parallel }_{2}^{2}.$ We interpret a lower error to indicate superior performance in these experiments.

In this simulation, GPSA’s alignment error decreased with more readout features and increased with greater distortion (Fig. 1c–e). GPSA achieved a substantially lower error than PASTE in all settings. This difference in error is largely due to the fact that PASTE applies a linear transformation and is unable to account for local nonlinear distortions. Furthermore, GPSA also outperforms PASTE with much larger numbers of spatial locations (Supplementary Fig. 2). These results imply that our model is robust to nonlinear warpings, distortions of different magnitudes and differences in the number of readout features.

#### Assessing alignment via readout prediction

Our experiments thus far have tested whether GPSA can align the spatial coordinates of distorted samples. However, we expect that similar expression patterns across aligned slices should colocalize within the estimated CCS. To test this, we attempted to predict held-out readout values using the posterior estimates of expression values localized within the CCS. In particular, we repeated the 2D experiment in the previous Section but held out 20% of the readout values from one of the slices. We then fit template-based GPSA and sampled the predicted expression values from the variational posterior predictive distribution at each CCS location:

$${\hat{f}}_{ij}^{{\,}(t)} \sim p({\mathrm{f}}_{ij}| {\hat{g}}_{i}^{(t)}),\quad {\hat{g}}_{i}^{(t)} \sim p({g}_{i}| {x}_{i}^{\star }),t=1,\cdots \,,T,$$

where *g* captures the CCS and *f* captures the noiseless distribution of the readouts at each location of the CCS. Because the posterior mean is not available in closed form, we approximate the posterior mean across *T* = 10 samples, ${\widehat{\rm{f}}}_{ij}=\frac{1}{T}\mathop{\sum }\nolimits_{t = 1}^{T}{\widehat{\rm{f}}}_{ij}^{{\,}(t)}$. We compute the MSE between the predictions and the true values. We compared GPSA to two baseline approaches: we fit a GP to each slice separately (‘separate’ GP), and we fit a GP to a concatenation of the slices (‘union’ GP). We found that GPSA achieves lower prediction error than the two baseline methods (Fig. 1f). This result suggests that GPSA estimates readout value distributions within a CCS that have excellent predictive capabilities.

### Estimating CCSs for ST

Having validated GPSA as a viable model for robust spatial alignment of high-dimensional observations, we next applied GPSA to spatially-resolved genomics data. Below, we present analyses of data collected from three technologies: ST^1^, the Visium platform^37^ and Slide-seqV2 (ref. ^3^). We also performed analyses with images of hematoxylin and eosin (H&E) stains jointly with the spatial genomics data (Supplementary Table 1).

For all datasets, we removed mitochondrial genes and spatial locations with low counts, normalized readouts at each spatial location by the total number of counts at that location and log transformed, centered and standardized gene counts. We further filtered the data to include genes with spatial variability (see Methods for details). The spatial locations for each slice were normalized such that both coordinates were in the interval (0, 10), as all slices were produced with approximately the same field of view.

#### Aligning ST profiles of breast cancer samples

We tested GPSA on an ST^1^ dataset made up of four slices of a breast cancer tumor (Supplementary Fig. 4).

We first validated GPSA on the ST data by perturbing the samples with an artificial warp and examining whether the CCS estimated by GPSA approximately removed the perturbation. For this experiment, we analyzed each of the four samples separately. We applied a synthetic GP warp (Methods) with *τ*^2^ = 0.5 and *ℓ*^2^ = 10 to each of the slices and ran de novo GPSA on these misaligned samples. For comparison, we ran PASTE and visualized the aligned coordinates for each method.

**Fig. 2:**
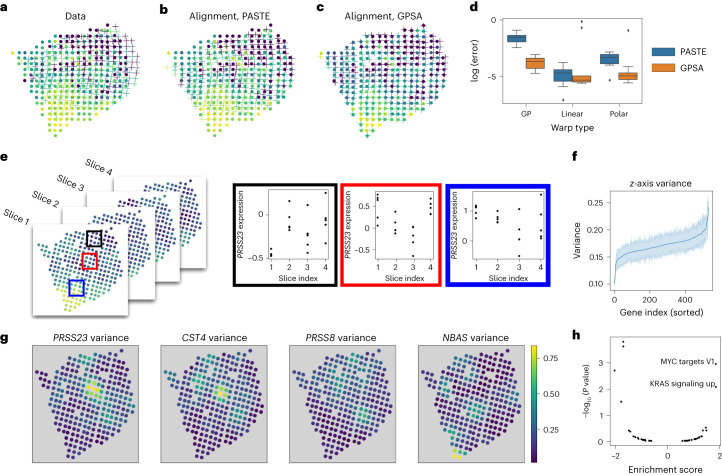
Aligning ST data from a breast cancer tumor. **a**, We applied a synthetic warp to one slice of the ST data^1^ to remove the spatial distortion. The original slice is plotted using dots, and the warped slice is plotted using crosses. Points are colored by the expression of one gene. **b**, Alignment from PASTE^25^, which applies a linear transformation. **c**, Alignment from GPSA. **d**, Alignment error (log scale) for three types of synthetic warps applied to the ST data. Results are shown for GPSA and PASTE; each method and warp was run ten times. Each box plot shows the minimum and the maximum values (whiskers), the median (center line) and the first and third quartiles (the box boundary); the outliers are plotted separately and removed from computation. **e**, Using the aligned spatial coordinates from GPSA of the four ST tumor slices, we estimated the variability of gene expression within each spatial location by computing the variance across slices for each gene. **f**, Spatial variance of each gene, averaged across spots, where the x axis has been sorted. **g**, Several genes show substantial variability across the slices. Points are colored by the estimated variance at each spatial location. **h**, Gene set enrichment analysis of the gene variance scores from **g**.

GPSA was able to recover the CCS (Fig. 2). Moreover, GPSA corrected the local distortions in the spatial coordinates. By contrast, PASTE’s global correction did not correct these distortions. To quantify the alignments, we computed the MSE between the aligned coordinates and the true coordinates for three types of synthetic warps: GP, linear and polar warps. We ran ten repetitions of each experiment. GPSA outperformed PASTE under the GP and polar warps and performed roughly the same as PASTE under linear warps (Fig. 2). This result suggests that GPSA robustly corrects local distortions on spatial coordinates.

#### Estimating expression variability across spatial locations

We next asked whether we could estimate the variability of gene expression at each spatial location. An accurate estimate of the variance would allow for useful analyses, such as understanding the spatial heterogeneity of gene expression in a particular tissue region and analyzing changes in expression along the *z* axis. Better alignment of the slices will lead to more precise quantification of variance.

We used the ST breast cancer data^1^ to quantify expression variation across slices. Here, we aligned all four slices using a 2D template-based alignment, using the second slice as the template. We then transferred each slice’s gene expression onto the template slice’s spatial coordinates by assigning each point in the template slice the average of its nearest neighbors in the corresponding aligned slice. Using these four slices within the CCS, we then computed the variance for each gene (Fig. 2e), representing a combination of experimental, biological and *z*-axis variability.

We found substantial variability in expression for several genes (Fig. 2f) that are related to tumor progression, including *PRSS23* (ref. ^38^) and *CST4* (ref. ^39^). To further investigate potential biological implications of this variation, we performed a gene set enrichment analysis, testing genes’ spatial variance (Fig. 2g) for over-representation or under-representation of specific gene modules. We found that ‘MYC targets’ and ‘upward KRAS signaling’, known to be associated with expression profiles in patients with breast cancer^40–43^, were enriched in genes with high estimated spatial variance. Thus, variability of expression across aligned slices at single locations highlights biologically informative markers.

#### Aligning samples in 3D space to create an atlas

Our analyses of the ST data thus far have ignored the 3D nature of the contiguous slices. Thus, we asked whether we could infer the third dimension (the *z* axis) to create a CCS plus localized expression distributions for the 3D tumor, what we would call a ‘3D tumor atlas’^30^.

To create a 3D tumor atlas, we fit GPSA on the four ST breast cancer slices, but we set the number of spatial dimensions to *D* = 3. We initialized the four slices’ *z*-axis coordinates as (0, 1, 2, 3) and allowed the model to warp these coordinates. Importantly, we used the same covariance function parameters for the warping GP across all spatial dimensions, which allows the alignment along the *z* axis to be informed by inferred spatial relationships along the *x* and *y* axes.

**Fig. 3:**
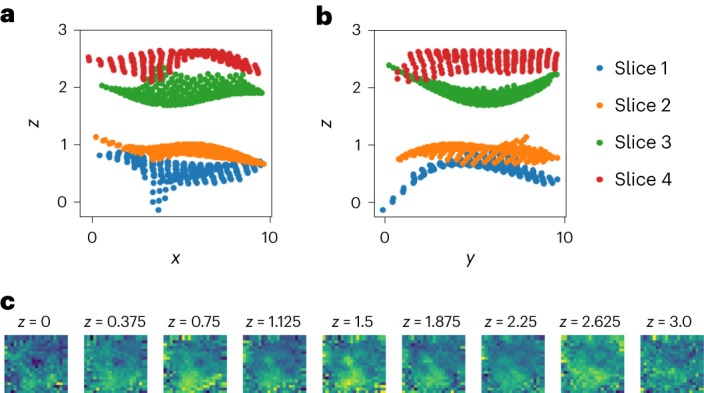
Three-dimensional alignment of ST breast cancer data. Aligned 3D coordinates plotted from two views. **a**,**b**, The *x* axis versus the *z* axis (**a**) and the *y* axis versus the *z* axis (**b**). Each image in **c** shows the imputed gene expression values for the gene *FN1* in one slice of the *z* axis. The location along the *z* axis increases from left to right and from top to bottom.

To perform the alignment, we used a two-step procedure. In the first step, we performed a template-based alignment with the second slice as the fixed template. In the second step, we fixed aligned coordinates from warped slices 1, 3 and 4 as the template and fit GPSA again, warping the second slice’s coordinates. This process resulted in a 3D CCS for the tumor, where we have an estimate of gene expression at each location in the 3D CCS. The aligned *z* axis showed substantial adjustments from the original positions (Fig. 3); we hypothesize that GPSA’s second-layer GP identified regions of the tumor that were functionally similar in terms of gene expression, and thus GPSA’s first-layer GP warped those spatial locations to be near one another within the CCS.

We imputed a dense 3D model of gene expression within the estimated CCS (Supplementary Fig. 5). We found that expression of specific genes varied smoothly across this space, with substantial variation along the *z* axis (Fig. 3). These findings suggest that GPSA is a feasible model for creating 3D atlases using spatial genomic slices from sequential samples from a single tissue.

#### Aligning Visium profiles of the mouse cortex

**Fig. 4:**
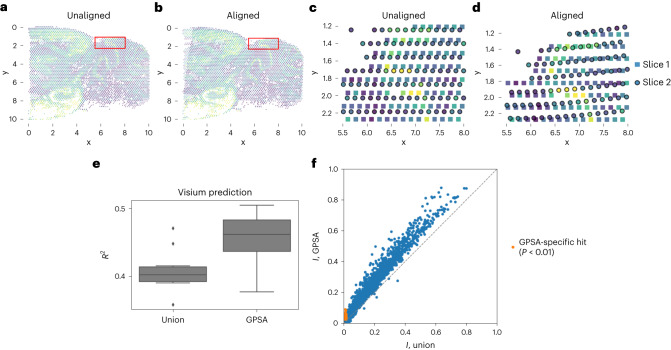
Aligning serial spatial gene expression slices from the mouse cortex from the Visium platform. We applied GPSA to two adjacent slices collected from the posterior–sagittal region of the mouse brain. **a**, Superposition of the two slices in the unaligned coordinate system. **b**, Superposition of the two slices in the aligned coordinate system. **c**, Magnified version of the region in the red bounding box in **a**. In the original slices, a cerebellar fold is misaligned (bottom left). **d**, Magnified version of the region in the red bounding box in **b**. The GPSA CCS corrects this distortion. **e**, Coefficient of determination (*R*^2^) for predictions of readout values versus ground truth on a held-out dataset. ‘Union’ represents predictions from a GP fit to a naive concatenation of the observed samples, and ‘GPSA’ refers to predictions from a GP fit jointly across all samples using the coordinate system. Each box plot shows the minimum and the maximum values (whiskers), the median (center line) and the first and third quartiles (the box boundary); the outliers are plotted separately and removed from computation (here, for ‘union’). **f**, Moran’s *I* statistic (measuring spatial correlation) for each gene under a union alignment and under GPSA’s alignment. Genes with BH-adjusted *P* values (*P* < 0.05) under GPSA but not under union are colored orange.

Next, we applied GPSA to data collected using the Visium platform from 10x Genomics^37^. These data (two adjacent slices) were collected from a cross-section of the sagittal–posterior region of the mouse brain. The slices contain measurements at 3,355 and 3,289 spatial locations. We again filtered the data, keeping spatially variable genes (Supplemental Methods 2.12) and leaving 135 genes. We fit template-based GPSA, designating the first sample as the template. In the original data, there was a small spatial mismatch in the cerebellar folds of the two slices (Fig. 4a,c). Examining the aligned coordinates, we found that GPSA was able to correct this distortion by adjusting the second slice downward to match the first slice (Fig. 4b,d).

To quantify the alignment, we fit GPSA using all of the spots from the first slice and 80% of the spots from the second slice, reserving the remaining 20% of the spots for testing. We then made predictions for expression levels at the held-out spots using two strategies: (1) naively stacking the two slices and making predictions using a GP (the union GP) and (2) using our estimated CCS and localized expression estimates to predict expression values. We computed *R*^2^ for the masked predictions, repeating this experiment five times for random train–test splits. Predictions using the aligned coordinates from GPSA outperformed those using the original coordinates (Fig. 4e).

We next asked whether downstream analyses of these data could be strengthened following alignment with GPSA. To do this, we tested our ability to identify spatially-correlated genes before and after alignment by computing Moran’s *I* score for each gene. We found that the scores were consistently higher following alignment with GPSA, and scores for several genes were statistically significant only after alignment (false discovery rate ≤ 0.1; Fig. 4f), indicating improved statistical power. Under the union alignment, we identified 2,644 (of 4,260) genes with spatial autocorrelation, while, under the GPSA alignment, we identified 2,945 genes with spatial autocorrelation (Benjamini–Hochberg (BH)-adjusted *P* < 0.1). Together, these findings imply that aligned coordinates strengthen downstream analyses of variation.

#### Aligning Slide-seqV2 profiles of the mouse hippocampus

We next leveraged a set of two tissue slices collected from the hippocampus region of two mice using Slide-seqV2^3^. These samples are not immediately comparable due to major shifts in the field of view (Supplementary Fig. 7). Thus, as a preprocessing step, we first applied a coarse manual rotation and translation to put the samples approximately within the same field of view. We fit GPSA to these slices using a template-based alignment with the first slice as the template.

**Fig. 5:**
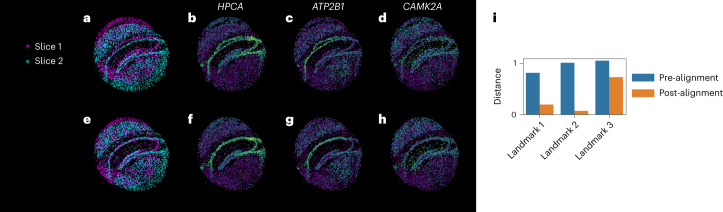
Alignment of Slide-seqV2 spatial gene expression profiles collected from the mouse hippocampus. Each plot in the top row shows a superposition of the two unaligned slices, and each plot in the bottom row shows a superposition of the aligned slices. **a**,**e** are colored by the slice identity, and **b**–**d**,**f**–**h** are colored by the expression levels of single genes (**b**,**f**, *HPCA*; **c**,**g**, *ATP2B1*; **d**,**h**, *CAMK2A*). **i**, The distance between the slices at three landmark locations before and after alignment with GPSA.

The aligned coordinates showed correspondence between the two slices for multiple major landmark regions. In particular, we found that the dentate gyrus and the CA1–CA3 pyramidal layer were well aligned (Fig. 5). Due to differences in the field of view and in the structure of the brains of two different mice, we did not expect to achieve a perfect one-to-one matching of the spatial coordinates. In particular, we observe that the choroid plexus was a prominent marker in the first slice but not in the second (Supplementary Fig. 8), and several other structures were not present in the second slice (Supplementary Fig. 9). The flexibility of GPSA allows for these distinctions. Moreover, a user could manually correct a deformation in the latent CCS if it were known to be incorrect (Discussion).

To further validate this CCS, we computed the distance between three landmark locations (two endpoints of the dentate gyrus and an edge of area CA3 (Supplementary Fig. 10)) in both slices before and after alignment. For all three landmarks, the distance between the slices decreased after alignment (Fig. 5i). This suggests that known structural landmarks are well aligned in GPSA even when hidden from the model.

Next, we fit GPSA while holding out a fraction of the spots and tested how well we could predict the expression values at the held-out spots before and after alignment. Again GPSA outperformed a concatenation of slices in prediction, and this improved prediction was largely consistent across genes (Supplementary Fig. 11).

### Multimodal alignment: incorporating histology images

We jointly aligned spatially-resolved gene expression and histology images. The histology images contain measurements for three color channels and thus are low dimensional relative to gene expression values; however, these images often contain interpretable features of anatomy widely used by pathologists, leading to their availability alongside spatial gene expression profiling. We hypothesize that including histology images in the alignment procedure may produce better alignments and enhance the interpretability of alignments in downstream analyses, such as enabling straightforward CCS annotation.

To test this hypothesis, we used mouse brain data from the Visium platform. For each slice, a histology image is pre-aligned to a Visium slice. We again fit template-based GPSA using the *S* = 2 slices with the first slice as the template, but this time we also include the histology images as phenotypic readout features and spatial locations.

**Fig. 6:**
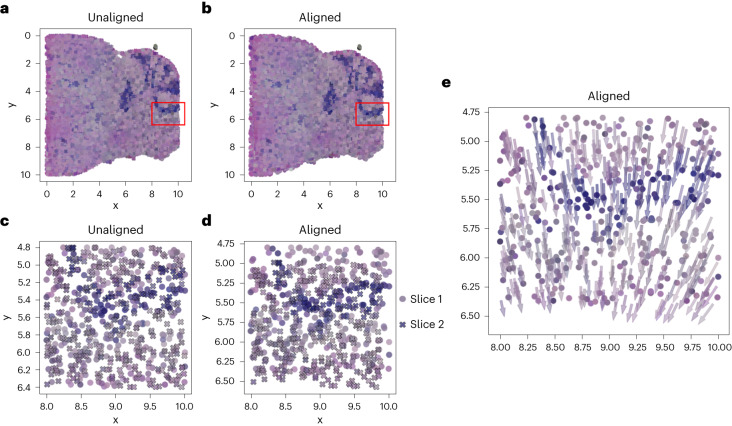
Joint alignment of spatial gene expression and histology images using mouse brain data from the Slide-seqV2 platform. **a**,**b**, A superposition of the histology images of the two slices in the unaligned and aligned coordinate systems, respectively. **c**,**d**, Magnified versions of the region in the red bounding box. **e**, The same data as **d**, but the points for the second slice are shown with arrows pointing in the direction of the alignment.

GPSA successfully aligned these multimodal samples (Fig. 6). In the original histology images, there was a slight misalignment in one of the cerebellar folds (Fig. 6c). After fitting GPSA, we observed that the alignment had been corrected (Fig. 6d). Examining gene expression in the corresponding region, we found that the darker histology region corresponded to higher levels of expression in the genes *CAMK2A* (BH-adjusted *P* value ≤ 1.0 × 10^−5^) and *MT-CO1* (BH-adjusted *P* value ≤ 5.0 × 10^−3^; Supplementary Fig. 12).

We computed a vector field showing the displacement of each spatial coordinate after the alignment. Substantial nonlinear warping was necessary to align the histology stains (Fig. 6e). These results suggest that GPSA may be used to align multimodal data including spatial gene expression and histology measurements, broadening its potential applications.

## Discussion

We have presented GPSA, a Bayesian two-layer GP model for aligning multiple spatial genomic and histology slices into a known or an unknown CCS. We have shown that our model can flexibly align samples from multiple spatial sequencing technologies, fields of view and data modalities. Current approaches such as PASTE^25^ and Splotch^26^ rely on linear transformations of spatial coordinates. We showed the necessity of allowing nonlinear warpings. We imagine a two-stage strategy for building tissue atlases: (1) running PASTE to find a coarse alignment and (2) running GPSA to tune the coarse alignment and produce a CCS with localized measurement distributions across the space. Given GPSA’s flexible assumptions, our model is applicable to many other spatial sequencing technologies (both present and future) with varying levels of resolution, fields of view and profiling.

Several future directions could be pursued. While we find that GPSA finds more accurate alignments than competing approaches, this comes at the cost of time. Our variational inducing point inference reduces the time complexity from *O*(*n*^3^) to *O*(*nm*^2^), where *n* and *m* are the number of readouts and inducing points, respectively. However, a large number of inducing points are often required for high-resolution technologies. Nonetheless, we find that the time required to fit GPSA to the data presented here is not prohibitive (Supplementary Fig. 13). Moreover, researchers only need to align their samples once with GPSA. Including known anatomical landmarks could speed up the alignment and lead to more biologically interpretable coordinate systems. To incorporate prior anatomical knowledge, structural landmarks could easily be included in the GPSA framework by fixing the annotated landmark locations in the CCS. However, an attractive feature of GPSA is its reliance on almost no prior knowledge about the structure of the tissue. Future work may also use landmarks inferred from the histology images to annotate the CCS in an automated manner. Finally, there remains an opportunity for a deeper theoretical study of GPSA and DGPs in general. Studying the posterior consistency of the kernel parameters and the latent variable **G** could lead to theoretical guarantees for the resulting CCSs.

## Methods

### Problem definition and notation

Here, we formalize the problem that we wish to solve. We define a spatially-resolved slice or slice from a spatial genomic or histology technology as a set of pairs ${\{({{{{\bf{x}}}}}_{i},{{{{\bf{y}}}}}_{i})\}}_{i = 1}^{n}$, where ${{{{\bf{x}}}}}_{i}\in {{\mathbb{R}}}^{D}$ is a vector of spatial coordinates encoding a single slice’s relative location in a D-dimensional space, and ${{{{\bf{y}}}}}_{i}\in {{\mathbb{R}}}^{p}$ is a vector of measured readout features at this location. Typically, *D* ∈ {2, 3, 4} in biomedical applications, where *D* = 4 corresponds to the spatiotemporal setting. We focus on *D* = {2, 3} in this paper. Following convention, we refer to a single location **x**_*i*_ as a spot, which may refer to a single cell, a subcellular location, a collection of cells or a single pixel depending on the technology. We arrange the observations from a slice into two data matrices: the spots’ relative locations ${{{\bf{X}}}}\in {{\mathbb{R}}}^{n\times D}$ and the phenotypic readouts associated with those spots ${{{\bf{Y}}}}\in {{\mathbb{R}}}^{n\times p}$.

To give concrete examples, in ST applications, **x**_*i*_ encodes a spatial location on a single tissue slice, and **y**_*i*_ is a vector of RNA transcript counts at this location for each of *p* genes. In a histology setting, **x**_*i*_ is a pixel location, and **y**_*i*_ is a vector containing the *p* image color channel readouts.

We assume that we have *S* spatially-resolved slices collected from the same tissue type and similar tissue region. Often, these slices will be adjacent slices from a single tissue, but, as we showed in our results, our approach is extensible to datasets collected from different tissue samples or individuals. Suppose slice *s* (*s* ∈ {1, …, *S*}) contains *n*_*s*_ spots, and let ${{{{\bf{X}}}}}^{s}={[{{{{\bf{x}}}}}_{1}^{s},{{{{\bf{x}}}}}_{2}^{s},\ldots ,{{{{\bf{x}}}}}_{{n}_{s}}^{s}]}^{\top }$ denote its spatial locations. Similarly, let ${{{{\bf{Y}}}}}^{s}={[{{{{\bf{y}}}}}_{1}^{s},{{{{\bf{y}}}}}_{2}^{s},\cdots ,{{{{\bf{y}}}}}_{{n}_{s}}^{s}]}^{\top }$ be the *s*th readout of feature values. We denote the total number of spots across slices as $N=\mathop{\sum }\nolimits_{s = 1}^{S}{n}_{s}$. We note that, in our framework, the slices may have different total numbers of spots and may be on different scales.

Our goal is to align these *S* slices’ spatial coordinates by creating a CCS such that the matching anatomical, structural and functional regions of each slice are mapped to the same absolute locations in the CCS. To do this, we seek correspondences between both the spatial coordinates and phenotypic readouts of each slice. Specifically, we seek *S* vector-valued warping functions *g*^1^, *g*^2^, …, *g*^*S*^, with ${g}^{s}:{{\mathbb{R}}}^{D}\to {{\mathbb{R}}}^{D}$, each of which maps a slice’s observed relative spatial coordinates into a shared CCS. Let ${{{{\bf{g}}}}}_{i}^{s}={g}^{s}({{{{\bf{x}}}}}_{i}^{s})$ denote evaluation of the *s*th warping function at spatial location ${{{{\bf{x}}}}}_{i}^{s}$. We call ${{{{\bf{g}}}}}_{i}^{s}$ the ‘aligned spatial location’ of this spot, and let the full set of aligned spatial locations be denoted as ${{{\bf{G}}}}=[{{{{\bf{g}}}}}_{1}^{1},{{{{\bf{g}}}}}_{2}^{1},\ldots ,{{{{\bf{g}}}}}_{i}^{s},{{{{\bf{g}}}}}_{i+1}^{s},\ldots ,{{{{\bf{g}}}}}_{{n}_{S}}^{S}]$.

Our goal is to estimate these warping functions ${\{{g}^{s}\}}_{s = 1}^{S}$ such that any two samples mapped to nearby points in the CCS, ${{{{\bf{g}}}}}_{i}^{s}\approx {{{{\bf{g}}}}}_{{i}^{{\prime} }}^{{s}^{{\prime} }}$, are structurally and functionally similar to one another. We consider three approaches that show the powerful behavior of our probabilistic model under uncertainty and censored information. First, we treat the multiple slices as biological replicates to leverage both spatial information and the measured readouts for alignment. Second, we consider multiple data modalities of the same biological system, assuming that the data come from approximately the same location in the absolute coordinate system to leverage the spatial locations and all modalities jointly. Third, we use multiple slices and infer their relationship along an unobserved *z* axis, assuming that the measured readouts vary across the *z* axis in a smooth manner. The flexibility of our GP framework allows each of these three approaches to alignment.

### Gaussian processes

A GP is a stochastic process defined as a collection of random variables in which any subset follows a multivariate Gaussian distribution. Specifically, *y*_1_, *y*_2_, … constitute a GP if, for any finite set of indices *i*_1_, *i*_2_, …, *i*_*n*_, it holds that

$${({y}_{{i}_{1}},{y}_{{i}_{2}},\cdots ,{y}_{{i}_{n}})}^{\top } \sim {{{{\mathcal{N}}}}}_{n}(\,{{{\boldsymbol{\mu }}}},{{{\boldsymbol{\Sigma }}}}),$$

where ***μ*** is a mean vector and **Σ** is a positive definite covariance matrix. GPs are widely used in functional data analysis, machine learning and spatial statistics due to their flexibility and expressiveness in modeling complex dependent data^44–48^. For example, in nonparametric regression models, GPs are commonly used to model unknown arbitrary functions; in Bayesian contexts, they act as priors over functions^49^.

GPs are often used as prior distributions over functions, as in this paper. In this case, for a function *f* defined on the domain ${{\mathbb{R}}}^{D}$, we denote a GP prior as

$$f \sim {{{\rm{GP}}}}(\mu ,k),$$

where $\mu :{{\mathbb{R}}}^{D}\to {\mathbb{R}}$ is a mean function and $k:{{\mathbb{R}}}^{D}\times {{\mathbb{R}}}^{D}\to {\mathbb{R}}$ is a positive definite covariance function (also known as a kernel function or covariogram). For noisy responses from the noiseless function *f*, we include Gaussian noise: $y \sim {{{\mathcal{N}}}}(f({{{\bf{x}}}}),{\sigma }^{2})$, where *σ*^2^ is often referred to as the ‘nugget’.

### Deep Gaussian processes

DGPs were developed to further extend the expressivity of GPs^50,51^. DGPs are a composition of functions, each of which is drawn from a GP. In the univariate case, the function drawn from an *L*-layer DGP is given by

$$f={f}_{L}\circ {f}_{L-1}\circ \cdots \circ {f}_{1},$$

where, for each *ℓ* = 1, …, *L*, we have ${f}_{\ell } \sim {{{\rm{GP}}}}({\mu }_{\ell },{k}_{\ell })$, and **y**_*ℓ*_ = *f*_*ℓ*_(*f*_*ℓ*−1_( ⋯ *f*_1_(**x**))) is the output of the *ℓ*th layer in an input sample **x**. In this work, we use two-layer DGPs or *L* = 2.

### Gaussian Process Spatial Alignment

#### First layer: warping functions

GPSA places GP priors on the warping functions *g*^1^, …, *g*^*S*^ that map the observed spatial coordinates onto a CCS. Focusing on the case with *D* = 2 spatial dimensions for demonstration, GPSA assumes that

$${{{{\bf{g}}}}}_{i}^{s}=\left[\begin{array}{c}{g}_{1}^{s}({{{{\bf{x}}}}}_{i}^{s})\\ {g}_{2}^{s}({{{{\bf{x}}}}}_{i}^{s})\end{array}\right],\quad {g}_{d}^{s} \sim {{{\rm{GP}}}}({\mu }_{gd},{k}_{g});\quad s=1,\ldots ,S,d=1,2,$$

where ${g}_{d}^{s}$ is the warping function for slice *s* for which the output is the *d*th spatial dimension, ${\mu }_{gd}:{{\mathbb{R}}}^{D}\to {\mathbb{R}}$ is a mean function, and ${k}_{g}:{{\mathbb{R}}}^{D}\times {{\mathbb{R}}}^{D}\to {\mathbb{R}}$ is a positive definite covariance function. We specify the mean of the aligned spatial location to be equal to the observed location, *μ*_*gd*_(**x**) = *x*_*d*_, which encourages the aligned coordinate for a given spatial location to be centered around the observed location. This assumption is useful to avoid extreme warps that drastically shift the mean of each observed location.

#### Second layer: modeling phenotypic readouts

GPSA then posits another set of functions ${\{{f}_{j}\}}_{j = 1}^{p}$ that describe the spatial organization of each phenotypic readout (for example, gene expression values) within the CCS. We place a GP prior on these functions as well. Letting ${y}_{ij}^{s}$ denote the value for feature *j* in spot *i* from slice *s*, GPSA assumes that

2 $${y}_{ij}^{s}={f}_{j}({{{{\bf{g}}}}}_{i}^{s})+\epsilon ,\quad {f}_{j} \approx {{{\rm{GP}}}}({\mu }_{f},{k}_{f}),\quad j=1,\ldots ,p,s=1,\ldots ,S,$$

where $\epsilon \sim {{{\mathcal{N}}}}(0,{\sigma }^{2})$ is a noise term, ${\mu }_{f}:{{\mathbb{R}}}^{D}\to {\mathbb{R}}$ is a mean function, and ${k}_{f}:{{\mathbb{R}}}^{D}\times {{\mathbb{R}}}^{D}\to {\mathbb{R}}$ is a positive definite covariance function. Let ${{{{\rm{f}}}}}_{ij}^{s}\in {\mathbb{R}}$ be the evaluation of *f*_*j*_ at input ${{{{\bf{g}}}}}_{i}^{s}$. We specify *μ*_*f*_ = 0, as we assume that the phenotypic readouts have been centered. Furthermore, let ${{{\bf{F}}}}=[{{{{\rm{f}}}}}_{11}^{1},{{{{\rm{f}}}}}_{21}^{1},\ldots ,{{{{\rm{f}}}}}_{ij}^{s},{{{{\rm{f}}}}}_{(i+1)j}^{s},\ldots ,{{{{\rm{f}}}}}_{{n}_{s}p}^{S}]$ denote the full set of function evaluations.

The above model results in a two-layer DGP where, for each slice *s*, the DGP is made up of a composition of two functions, *f*∘*g*^*s*^.

### Posterior inference for the common coordinate system

We have two statistical objectives with the GPSA model: estimating the CCS, as represented by the latent variable **G**, and estimating the warped, denoised and localized values for the phenotypic readouts for each slice, represented by **F**. The CCS gives us an atlas of the system; the warped and smoothed readouts may be used for downstream analysis of the aligned slices. Thus, in our Bayesian GPSA framework, the posterior distribution of interest is

3 $$p({{{\bf{G}}}},{{{\bf{F}}}}| {{{\bf{X}}}},{{{\bf{Y}}}},\Theta )=\frac{p({{{\bf{X}}}},{{{\bf{Y}}}}| {{{\bf{G}}}},{{{\bf{F}}}},\Theta )p({{{\bf{G}}}},{{{\bf{F}}}})}{Z},$$

where the vector Θ contains the parameters for the mean and covariance functions and *Z* = *p*(**X**, **Y**∣Θ) is a normalizing constant. However, *Z* is analytically intractable in DGPs^50^. Thus, we use stochastic variational inference with inducing variables to approximate the posterior distribution over **G** and **F** (ref. ^52^).

### Stochastic variational inference for GPSA

Although closed-form posterior distributions are available in GPs, this is not the case in DGPs. To perform approximate inference, we leverage a sparse GP framework using inducing points^51,53,54^. Because GPSA is a two-layer DGP, we include inducing points at each of the two layers. In particular, suppose we have a set of *M*^*s*^ < *n*_*s*_ inducing locations (also known as pseudo-inputs) for each slice ${\widetilde{{{{\bf{X}}}}}}^{1},\ldots ,{\widetilde{{{{\bf{X}}}}}}^{S}\in {{\mathbb{R}}}^{{M}^{s}\times D}$ and another set of *M* < *N* inducing locations in the CCS layer $\widetilde{{{{\bf{G}}}}}\in {{\mathbb{R}}}^{M\times D}$. We then denote the associated set of inducing values (pseudo-outputs) for the two layers as ${{{{\bf{U}}}}}^{{G}^{1}},\ldots ,{{{{\bf{U}}}}}^{{G}^{S}}\in {{\mathbb{R}}}^{{M}^{s}\times D}$ and ${{{{\bf{U}}}}}^{F}\in {{\mathbb{R}}}^{M\times p}$, respectively. The joint model (omitting dependence on the covariance function parameters Θ) is then

4 $$\begin{array}{l}p({\mathbf{G}}, {\mathbf{F}}, {\mathbf{U}}^G, {\mathbf{U}}^F, {\mathbf{X}}, {\mathbf{Y}}) =\\ \underbrace{p({\mathbf{Y}} | {\mathbf{F}})}_{{\rm{Noise}}\,{\rm{model}}} \, \underbrace{p({\mathbf{F}} | {\mathbf{U}}^F, {\mathbf{G}})}_{{\rm{Readout}}\,{\rm{GP}}} \, \underbrace{p({\mathbf{U}}^F | {\widetilde{\mathbf{G}}})}_{{\rm{Inducing}}\,{\rm{prior}}} \, \underbrace{p({\mathbf{G}} | {\mathbf{U}}^G, {\mathbf{X}})}_{{\rm{Warp}}\,{\rm{GP}}} \, \underbrace{p({\mathbf{U}}^G | {\widetilde{\mathbf{X}}})}_{{\rm{Inducing}}\,{\rm{prior}}}.\end{array}$$

Note that *p*(**F**∣**U**^*F*^, **G**) and *p*(**G**∣**U**^*G*^, **X**) have closed forms because they are conditional multivariate Gaussians. If a Gaussian noise model is assumed, then *p*(**Y**∣**F**) also has a closed form. However, inference in this model scales cubically with the number of spots; therefore, we seek a faster variational approach.

We now specify a variational model *Q*, with parameters that we will optimize to approximate the exact posterior (equation (3)). Following earlier work^50^, we use the following form for the approximate posterior:

5 $$Q=p({{{\bf{F}}}}| {{{{\bf{U}}}}}^{F},{{{\bf{G}}}})q({{{{\bf{U}}}}}^{F})p({{{\bf{G}}}}| {{{{\bf{U}}}}}^{G},{{{\bf{X}}}})q({{{{\bf{U}}}}}^{G}),$$

where *q*(**U**^*F*^) and *q*(**U**^*G*^) are chosen to be multivariate normal distributions. We denote the variational parameters collectively as *ϕ*. Because all distributions are Gaussian, we can analytically marginalize out the pseudo-outputs **U**^*F*^ and **U**^*F*^ (ref. ^51^). See Appendix for details.

The optimization problem is then to minimize the Kullback Leibler (KL) divergence from the exact posterior (equation (3)) to the approximate posterior (equation (5)) with respect to the variational parameters. This is equivalent to maximizing a lower bound on the log marginal likelihood ${{{\mathcal{L}}}}\le \log p({{{\bf{Y}}}})$ (the evidence lower bound or ELBO). The variational parameters *ϕ* are made up of the parameters of the prior distributions for the pseudo-outputs *q*(**U**^*F*^) and *q*(**U**^*G*^) and optionally the inducing locations ${\widetilde{{{{\bf{X}}}}}}^{1},\ldots ,{\widetilde{{{{\bf{X}}}}}}^{S},\widetilde{{{{\bf{G}}}}}$. More precisely, our optimization problem is

6 $$\mathop{\max }\limits_{\phi }{{{\mathcal{L}}}},\quad {{{\mathcal{L}}}}={{\mathbb{E}}}_{Q}\left[\log \frac{p({{{\bf{Y}}}}| {{{\bf{F}}}})p({{{{\bf{U}}}}}^{F}| \widetilde{{{{\bf{G}}}}})p({{{{\bf{U}}}}}^{G}| \widetilde{{{{\bf{X}}}}})}{{q}_{\phi }({{{{\bf{U}}}}}^{F}){q}_{\phi }({{{{\bf{U}}}}}^{G})}\right].$$

We provide a complete derivation and explanation of this lower bound in the next section. Although this lower bound cannot be evaluated in closed form, we can efficiently sample from it and use these samples to maximize with respect to the variational parameters *ϕ*.

### Maximizing the ELBO in GPSA

Recall that the ELBO for a generic model with observed data *x*, latent variable *z* and approximating distribution *q* is given by

$${{{\mathcal{L}}}}={{\mathbb{E}}}_{q(z)}\left[\log \frac{p(x,z)}{q(z)}\right],$$

where *p*(*x*, *z*) is the joint model density, and *q*(*z*) is the variational distribution.

Plugging in our GPSA model, the ELBO is given by
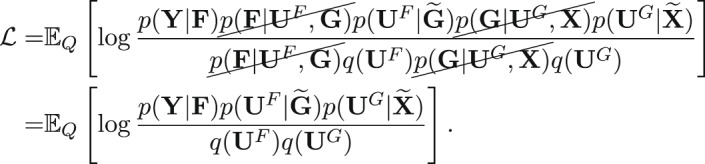


We can split equation (6) into a term containing the expected log likelihood and two terms that are KL divergences:

7 $${{{\mathcal{L}}}}={{\mathbb{E}}}_{Q}\left[\log p({{{\bf{Y}}}}| {{{\bf{F}}}})\right]-{{\mathbb{E}}}_{Q}\left[\log \frac{q({{{{\bf{U}}}}}^{F})}{p({{{{\bf{U}}}}}^{F}| \widetilde{{{{\bf{G}}}}})}\right]-{{\mathbb{E}}}_{Q}\left[\log \frac{q({{{{\bf{U}}}}}^{G})}{p({{{{\bf{U}}}}}^{G}| \widetilde{{{{\bf{X}}}}})}\right]$$

8 $$={{\mathbb{E}}}_{Q}\left[\log p({{{\bf{Y}}}}| {{{\bf{F}}}})\right]-{D}_{\rm{KL}}\left(q({{{{\bf{U}}}}}^{F})\parallel p\left({{{{\bf{U}}}}}^{F}| \widetilde{{{{\bf{G}}}}}\right.\right)-{D}_{\rm{KL}}\left(q({{{{\bf{U}}}}}^{G})\parallel p({{{{\bf{U}}}}}^{G}| \widetilde{{{{\bf{X}}}}})\right).$$

Because we let *q*(**U**^*F*^) and *q*(**U**^*G*^) be multivariate Gaussians, the KL divergence has a closed form, and the only remaining term to estimate is the expected log likelihood (the first term in equation (8)). We estimate this term with a Monte Carlo approximation. Given *T* samples of **F**, our estimate is

$${{\mathbb{E}}}_{Q}\left[\log p({{{\bf{Y}}}}| {{{\bf{F}}}})\right]\sim \frac{1}{T}\mathop{\sum }\limits_{t=1}^{T}\log p({{{\bf{Y}}}}| {\widehat{{{{\bf{F}}}}}}_{t}),$$

where ${\widehat{{{{\bf{F}}}}}}_{1},\ldots ,{\widehat{{{{\bf{F}}}}}}_{T} \approx Q$. We use a two-stage procedure to obtain these samples. First, we draw samples of $\widehat{{{{\bf{G}}}}}$ from $p({{{\bf{G}}}}| {{{\bf{X}}}},\widetilde{{{{\bf{X}}}}})=\int\,p({{{\bf{G}}}}| {{{{\bf{U}}}}}^{G},{{{\bf{X}}}},\widetilde{{{{\bf{X}}}}})p({{{{\bf{U}}}}}^{G}| \widetilde{{{{\bf{X}}}}})d{{{{\bf{U}}}}}^{G}.$ Second, we draw samples of $\widehat{{{{\bf{F}}}}}$ from $p({{{\bf{F}}}}| \widehat{{{{\bf{G}}}}},\widetilde{{{{\bf{G}}}}})=\int\,p({{{\bf{F}}}}| {{{{\bf{U}}}}}^{F},{{{\bf{G}}}},\widetilde{{{{\bf{G}}}}})p({{{{\bf{U}}}}}^{F}| \widetilde{{{{\bf{G}}}}})d{{{{\bf{U}}}}}^{F}.$ We can write each of these distributions in closed form.

Let $q({{{{\bf{u}}}}}_{d}^{Gs})={{{\mathcal{N}}}}({{{{\bf{m}}}}}_{d}^{Gs},{{{{\bf{S}}}}}_{d}^{Gs}).$ The marginal for **G** is given by

$$q\left({{{{\bf{g}}}}}_{d}^{s}| {{{{\bf{m}}}}}_{d}^{Gs},{{{{\bf{S}}}}}_{d}^{Gs};{{{\bf{X}}}},\widetilde{{{{\bf{X}}}}}\right)={{{\mathcal{N}}}}\left({\widetilde{\mu }}_{d}^{Gs},{\widetilde{\Sigma }}_{d}^{Gs}\right)$$

with

$${\widetilde{\mu }}_{d}^{Gs}=m({{{{\bf{X}}}}}^{s})+{{{{\bf{K}}}}}_{{\widetilde{{{{\bf{X}}}}}}^{s}{{{{\bf{X}}}}}^{s}}^{\top }{{{{\bf{K}}}}}_{{\widetilde{{{{\bf{X}}}}}}^{s}{\widetilde{{{{\bf{X}}}}}}^{s}}^{-1}({{{{\bf{m}}}}}_{d}^{Gs}-m({\widetilde{{{{\bf{X}}}}}}^{s}))$$

$${\widetilde{\Sigma }}_{d}^{Gs}={{{{\bf{K}}}}}_{{{{{\bf{X}}}}}^{s}{{{{\bf{X}}}}}^{s}}-{{{{\bf{K}}}}}_{{\widetilde{{{{\bf{X}}}}}}^{s}{{{{\bf{X}}}}}^{s}}^{\top }{{{{\bf{K}}}}}_{{\widetilde{{{{\bf{X}}}}}}^{s}{\widetilde{{{{\bf{X}}}}}}^{s}}^{-1}({{{{\bf{K}}}}}_{{\widetilde{{{{\bf{X}}}}}}^{s}{\widetilde{{{{\bf{X}}}}}}^{s}}-{{{{\bf{S}}}}}_{d}^{Gs}){{{{\bf{K}}}}}_{{\widetilde{{{{\bf{X}}}}}}^{s}{\widetilde{{{{\bf{X}}}}}}^{s}}^{-1}{{{{\bf{K}}}}}_{{\widetilde{{{{\bf{X}}}}}}^{s}{{{{\bf{X}}}}}^{s}}.$$

Let $q({{{{\bf{u}}}}}_{j}^{F})={{{\mathcal{N}}}}({{{{\bf{m}}}}}_{j}^{F},{{{{\bf{S}}}}}_{j}^{F}).$ The marginal for **F** is given by

$$q\left({{{{\bf{f}}}}}_{j}| {{{{\bf{m}}}}}_{j}^{F},{{{{\bf{S}}}}}_{j}^{F};{{{\bf{G}}}},\widetilde{{{{\bf{G}}}}}\right)={{{\mathcal{N}}}}\left({\widetilde{\mu }}_{j}^{F},{\widetilde{\Sigma }}_{j}^{F}\right)$$

with

9 $${\widetilde{\mu }}_{j}^{F}=m({{{\bf{G}}}})+{{{{\bf{K}}}}}_{\widetilde{{{{\bf{G}}}}}{{{\bf{G}}}}}^{\top }{{{{\bf{K}}}}}_{\widetilde{{{{\bf{G}}}}}\widetilde{{{{\bf{G}}}}}}^{-1}({{{{\bf{m}}}}}_{j}^{F}-m(\widetilde{{{{\bf{G}}}}}))$$

10 $${\widetilde{\Sigma }}_{j}^{F}={{{{\bf{K}}}}}_{{{{\bf{G}}}}{{{\bf{G}}}}}-{{{{\bf{K}}}}}_{\widetilde{{{{\bf{G}}}}}{{{\bf{G}}}}}^{\top }{{{{\bf{K}}}}}_{\widetilde{{{{\bf{G}}}}}\widetilde{{{{\bf{G}}}}}}^{-1}({{{{\bf{K}}}}}_{\widetilde{{{{\bf{G}}}}}\widetilde{{{{\bf{G}}}}}}-{{{{\bf{S}}}}}_{j}^{F}){{{{\bf{K}}}}}_{\widetilde{{{{\bf{G}}}}}\widetilde{{{{\bf{G}}}}}}^{-1}{{{{\bf{K}}}}}_{\widetilde{{{{\bf{G}}}}}{{{\bf{G}}}}}.$$

We then maximize the ELBO with respect to the variational parameters, as well as the covariance function parameters. If the covariance function parameters are optimized, one can regularize the covariance function parameters to avoid unrealistic warping functions. Under our Bayesian framework, we can place a prior distribution on the covariance function parameters to limit the warps to be small and stable.

This procedure is also amenable to stochastic optimization algorithms^52^. In terms of memory consumption, GPSA is extremely scalable. In particular, stochastic optimization algorithms open the door to scale GPSA to datasets with millions of spots by using a subset of the spots (a ‘mini-batch’) on each iteration of optimization. The required memory consumption will thus scale with the chosen mini-batch size, which can be made arbitrarily small depending on a user’s memory constraints.

### Multivariate correlated outcomes

In its simplest form, GPSA assumes that feature readouts are independent of one another by modeling each with a separate GP-distributed function *f*_*j*_. However, given that our phenotypic readouts of interest (gene expression, for example) are often highly correlated between features, we would like to leverage the correlation between readouts to fit *f*. There are several approaches to accounting for this correlation^35,55,56^.

We choose to leverage the LMC^35^. Rather than allowing *p* separate GPs, the LMC assumes that there are *L* < *p* latent GPs and that the observed readouts are a linear combination of the outputs of these latent GPs. To incorporate this into our registration model, we assume the following model for the second-layer GP:

$$\begin{array}{rcl}{{{{\bf{y}}}}}_{i}^{s}&=&{{{\bf{W}}}}{{{\bf{F}}}}+\epsilon \\ {\left[{{{\bf{F}}}}\right]}_{\ell i}={f}_{\ell }({{{{\bf{g}}}}}_{i})& \sim &{{{\rm{GP}}}}({\mu }_{\ell }({{{{\bf{g}}}}}_{i}),{k}_{\ell }({{{{\bf{g}}}}}_{i},{{{\bf{G}}}}))\\ \epsilon & \sim &{{{\mathcal{N}}}}({{{\bf{0}}}},{\sigma }^{2}{{{\bf{I}}}})\end{array}$$

where ${{{\bf{W}}}}\in {{\mathbb{R}}}^{p\times L}$ is a loading matrix, ${{{\bf{F}}}}\in {{\mathbb{R}}}^{L\times N}$ is a matrix containing latent factors and I is the identity matrix. Given a set of warped coordinates **G**, our likelihood is then

$$\begin{array}{rcl}p({{{\bf{Y}}}},{{{\bf{F}}}}| {{{\bf{W}}}},{{{\bf{G}}}},\theta ,{\sigma }^{2})&=&\mathop{\prod }\limits_{j=1}^{p}p({{{{\bf{y}}}}}_{j}| {{{\bf{F}}}},{{{{\bf{w}}}}}_{j},{\sigma }^{2})\mathop{\prod }\limits_{l=1}^{L}p({{{{\bf{f}}}}}_{l}| {{{\bf{G}}}})\\ &=&\mathop{\prod }\limits_{j=1}^{p}{{{\mathcal{N}}}}({{{{\bf{y}}}}}_{j}| {{{\bf{F}}}}{{{{\bf{w}}}}}_{j},{\sigma }^{2}{I}_{N})\mathop{\prod }\limits_{l=1}^{L}{{{\mathcal{N}}}}({{{{\bf{f}}}}}_{l}| {\mu }_{l}({{{\bf{G}}}}),{K}_{{{{\bf{G}}}}{{{\bf{G}}}}}).\end{array}$$

Including the warp model, our entire joint model becomes

$$\begin{array}{l}p({\mathbf{Y}}, {\mathbf{F}}, {\mathbf{G}} | {\mathbf{W}}, \theta, \sigma^2) =\\ \underbrace{\prod\limits_{j=1}^p {\mathcal{N}}({\mathbf{y}}_j | {\textbf{F}} {\mathbf{w}}_j, \sigma^2 I_N)}_{{\text{Noise}}} \underbrace{\prod\limits_{l=1}^L {\mathcal{N}}({\mathbf{f}}_l | \mu_l({\mathbf{G}}), K_{{\mathbf{G}} {\mathbf{G}}})}_{{\text{LMC}}} \underbrace{\prod\limits_{s=1}^S \prod\limits_{d=1}^D {\mathcal{N}}({\textbf{g}}_{d}^s | \mu_{d}^s({\mathbf{X}}^s), {\mathbf{K}}_{{\mathbf{X}}^s {\mathbf{X}}^s})}_{{\rm{Warp}}\,{\rm{prior}}}.\end{array}$$

In our applications, we may not be interested in directly estimating the latent factors **F**. We can marginalize these out^57^ and write the likelihood as

$${{{\rm{vec}}}}({{{\bf{Y}}}})| {{{\bf{G}}}} \sim {{{\mathcal{N}}}}(\mu ,\Sigma ),$$

where

$$\mu ={{{{\bf{0}}}}}_{Np},\quad \Sigma =\mathop{\sum }\limits_{l=1}^{L}{K}_{{{{\bf{G}}}}{{{\bf{G}}}}}^{(l)}\otimes {{{{\bf{w}}}}}_{l}{{{{\bf{w}}}}}_{l}^{\top }.$$

If the latent covariance functions *k*_1_, …, *k*_*L*_ are the same, the covariance simplifies as Σ = *K*_**GG**_ ⊗ **WW**^⊤^.

### Multimodal outcomes

We may sometimes have access to multiple samples from each slice, each of whose phenotypic readouts are collected from different modalities. For example, we may have an ST sample and a histology image in each slice. While both of these modalities lie in a 2D spatial coordinate system, they have different response values. In this example, the ST readouts will be ${{{{\bf{Y}}}}}^{1}\in {{\mathbb{R}}}^{n\times p}$, where *p* is the number of genes, while the histology image readouts will be ${{{{\bf{Y}}}}}^{2}\in {{\mathbb{R}}}^{m\times q}$, where *q* is the number of color channels.

Our model can easily accommodate this setting. We assume that the different modalities are already aligned within each slice, which is a reasonable assumption in practice. Instead of computing the likelihood for only one set of phenotypic readouts, we compute it for each modality’s phenotypic readouts. For example, the likelihood becomes

$$\begin{array}{rcl}p({{{\bf{Y}}}},{{{\bf{G}}}}| {{{\bf{X}}}},\theta ,{\sigma }^{2})&=&p({{{\bf{Y}}}}| {{{\bf{G}}}},{\theta }_{o},{\sigma }^{2})p({{{\bf{G}}}}| {{{\bf{X}}}},{\theta }_{w})\\ &=&\mathop{\prod }\limits_{m=1}^{M}\mathop{\prod }\limits_{j=1}^{{p}_{m}}p({{{{\bf{y}}}}}_{j}^{m}| {{{{\bf{G}}}}}^{m},{\theta }_{o},{\sigma }^{2})\mathop{\prod }\limits_{s=1}^{S}\mathop{\prod }\limits_{d=1}^{D}p({{{{\bf{g}}}}}_{d}^{s}| {{{{\bf{X}}}}}^{s},{\theta }_{w})\end{array},$$

where *M* is the number of modalities, *p*_*m*_ is the number of readout features in modality *m*, **Y**^*m*^ is the set of readout features for modality *m*, and σ2 is the variance of the Gaussian noise of each readout.

### Non-Gaussian likelihoods

We can accommodate non-Gaussian likelihoods in this model. In particular, we can specify the likelihood in equation (4), *p*(**Y**∣**F**), to be any suitable data likelihood. In the setting of sequencing data, the measurements often come in the form of nonnegative integer counts, for which a Poisson likelihood is often a reasonable choice.

### Imputing dense spatial readouts under GPSA

The second layer of GPSA represents a mapping from the CCS to the observed phenotypic readouts. Thus, for any location in the CCS (regardless of whether a sample location is mapped to this point in the first layer or not), we can compute the variational parameters for the phenotypic readouts at this location (equations (9) and (10)). This allows for querying across a dense grid of locations in the CCS, yielding a distribution over the phenotypic readouts at these locations.

### Model settings and preprocessing for experiments

In our experiments, we normalize all spatial coordinates so that the minimum *x* and *y* coordinate values are 0, and the maximum coordinate values are 10.

For all experiments, we specify the mean function of the GP prior for the warping functions to be the identity function. This choice is motivated by our expectation that most distortions in tissue samples will be relatively small and local, with large translations between slices being uncommon. We use the RBF covariance function for the first-layer GPs. The RBF covariance function is given by

11 $$k(x,{x}^{{\prime} })={\tau }^{2}\exp \left\{-\frac{{(x-{x}^{{\prime} })}^{2}}{{\ell }^{2}}\right\},$$

where *ℓ* is the length scale parameter, and *τ*^2^ is the spatial variance parameter. Intuitively, *ℓ* controls how different the warping function is locally, and *τ*^2^ controls the overall magnitude of the warping function (Supplementary Fig. 5). For the second layer of the multi-output GP, with an LMC covariance function, we infer the covariance function parameters using maximum likelihood. Model parameters, including covariance function parameters, are fitted during training by maximizing a lower bound on the log marginal likelihood of the data. For the first-layer GP (the warp GP) in de novo alignments, we fix the covariance function parameters before model fitting. Specifically, we fix the length scale as *ℓ* = 10 and the spatial variance as *σ*^2^ = 1 to ensure smooth and minimal warps. We found that these choices are relatively robust within a range (Supplementary Fig. 18). Our empirical tests show that the model’s performance tends to be stable for higher values of *σ*^2^ and *ℓ*. This is likely due to the fact that the model is more constrained with lower values of *σ*^2^ and *ℓ* (that is, it is difficult for the model to accommodate distortions with large magnitude under these parameter settings).

For our applications to spatial genomics data, we filter the readout features to features that show spatial correlation. Specifically, for each readout feature, we compute Moran’s *I* statistic^58^ (Supplementary Fig. 19) and retain features in the top 5% of *I* scores. We find that this approach identifies genes with high spatial variability (Supplementary Figs. 16 and 17) and identifies genes that were identified in previous work^15^. More complicated procedures to identify spatially variable genes could be used^15^, but this is not the primary focus of our work. This step increases the efficiency of GPSA not only by reducing the dimension of the readout features but also by removing features that are not correlated across space and would not aid a spatial alignment (Supplementary Fig. 8).

### Synthetic warps

Throughout our experiments, we apply three different types of random warps, which we describe here.

1. Linear warp: this warp applies a linear transformation to the observed spatial coordinates for each slice **X**^*s*^ such that ${\widetilde{x}}_{d}^{s}={({{{{\bf{x}}}}}^{s})}^{\top }{{{{\boldsymbol{\beta }}}}}_{d}^{s}+{\beta }_{d_0}^{s}+\epsilon$ for *d* ∈ {1, …, *D*}, where ${{{{\boldsymbol{\beta }}}}}_{d}^{s}\in {{\mathbb{R}}}^{D},{\beta }_{d_0}^{s}\in {\mathbb{R}}$ are the slope and intercept, respectively, and $\epsilon \sim {{{\mathcal{N}}}}(0,{\sigma }^{2})$ is a noise term.
2. Polar warp: for a single spatial sample to be represented as ${{{\bf{x}}}}={[{x}_{1},{x}_{2}]}^{\top }$, this function is defined as
  $${g}^{s}(x;\theta )=\left[\begin{array}{c}{x}_{1}+r\cos \phi \\ {x}_{2}+r\sin \phi \end{array}\right],$$
  where *θ* = {*r*, *ϕ*}. We further parametrize *θ* to allow for location-specific distortions. Thus, *θ* is implicitly a function of *x* as well,
  $$\left[\begin{array}{c}{r}_{x}\\ {\phi }_{x}\end{array}\right]=\theta (x)={{{\bf{B}}}}{{{\bf{x}}}},$$
  where **B** is a 2 × 2 coefficient matrix. The full warping function can then be written as
  $$\begin{array}{rcl}{g}^{s}(x;\theta )=\left[\begin{array}{c}{x}_{1}+{b}_{11}{x}_{1}\cos ({b}_{12}{x}_{1})\\ {x}_{2}+{b}_{21}{x}_{1}\sin ({b}_{22}{x}_{2})\end{array}\right].\end{array}$$
3. GP warp applies a transformation function that is drawn from a GP:
  12 $${\widetilde{{{{\bf{x}}}}}}_{d}^{s}={f}_{d}^{\,s}({{{{\bf{x}}}}}_{d}^{s})+\epsilon ,\quad {f}_{d}^{\,s}({{{{\bf{x}}}}}_{d}^{s}) \sim {{{\rm{GP}}}}({{{{\bf{x}}}}}_{d}^{s},{{{{\bf{K}}}}}_{{{{{\bf{x}}}}}_{d}^{s}{{{{\bf{x}}}}}_{d}^{s}}).$$

### Reporting summary

Further information on research design is available in the Nature Portfolio Reporting Summary linked to this article.

## Online content

Any methods, additional references, Nature Portfolio reporting summaries, source data, extended data, supplementary information, acknowledgements, peer review information; details of author contributions and competing interests; and statements of data and code availability are available at 10.1038/s41592-023-01972-2.

## Supplementary information

Supplementary Information Supplementary Figs. 1–23 and Table 1

Reporting Summary

## Data Availability

The following data are available: (1) ST data were obtained from the PASTE code repository: https://github.com/raphael-group/paste. All four layers from the ‘sample_data/’ directory were used. (2) Visium data were obtained from the 10x Genomics website. Data for the two slices were downloaded from the ‘Datasets’ page. Specifically, spatial gene expression and hematoxylin and eosin stains were downloaded from the following links: mouse brain serial section 1 (sagittal–posterior; https://www.10xgenomics.com/resources/datasets/mouse-brain-serial-section-1-sagittal-posterior-1-standard-1-1-0) and mouse brain serial section 2 (sagittal–posterior; https://www.10xgenomics.com/resources/datasets/mouse-brain-serial-section-2-sagittal-posterior-1-standard-1-1-0). (3) Slide-seqV2 data were downloaded from the Broad Institute’s Single Cell Portal: https://singlecell.broadinstitute.org/single_cell/study/SCP815/highly-sensitive-spatial-transcriptomics-at-near-cellular-resolution-with-slide-seqv2. Two pucks corresponding to the mouse hippocampus were used: Puck_191204_01 and Puck_200115_08.

## Code availability

Code for the model and experiments is available at https://github.com/andrewcharlesjones/spatial-alignment. All experiments in the paper can be run with the Python scripts in the ‘experiments/’ directory of the GitHub repository.
