# Supplementary material for: Alignment of spatial genomics data using deep Gaussian processes
Source: Nat Methods. 2023 Aug 17;20(9):1379–87. doi: 10.1038/s41592-023-01972-2 (PMC10482692; doi:10.1038/s41592-023-01972-2)
Supplement: Supplementary file 1 — Supplementary Figs. 1–23 and Table 1 [file 41592_2023_1972_MOESM1_ESM.pdf]

nature methods

Article https://doi.org/10.1038/s41592-023-01972-2

Alignment of spatial genomics data using deep Gaussian processes

In the format provided by the authors and unedited

Supplementary materials for *Alignment of spatial genomics data using deep Gaussian processes*

Andrew Jones[1], F. William Townes[2], Didong Li[3], and Barbara E. Engelhardt[4,5†]
[1]*Department of Computer Science, Princeton University*
[2]*Department of Biostatistics, University of North Carolina, Chapel Hill*
[3]*Department of Statistics and Data Science, Carnegie Mellon University*
[4]*Gladstone Institutes*
[5]*Department of Biomedical Data Science, Stanford University*

Supplementary Information

Time complexity

Running GPSA for $5,000$ epochs on the Visium mouse brain dataset with $M^s = 200, M = 200$ took 208 minutes. Running GPSA for $5,000$ epochs on the Slide-seqV2 mouse hippocampus dataset with $M^s = 200, M = 200$ took 774 minutes.

Aligning samples with different fields-of-view

In practice, the overlap in spatial coverage of samples may not be perfect. There may be settings in which we wish to align two slices whose fields-of-views are different. To simulate this scenario, we generated data in which one slice was generated from a grid of spatial coordinates as before, and a second slice that was a small window fully contained within the original spatial coordinates and on the same spot grid (Figure S1a). We then fit *template-based* GPSA, using the larger slice as the template, to these two slices and extracted the aligned coordinates. For comparison, we also ran PASTE and compared results with those from GPSA.

[†]Current affiliation: Department of Statistics and Data Science, Carnegie Mellon University
[††]Current affiliation: Department of Biostatistics, University of North Carolina at Chapel Hill
[†††]Current affiliation: Gladstone Institutes and Department of Biomedical Data Science, Stanford University

GPSA recovers the alignment between two slices even when the fields-of-view are different (Figure S1a-c). As above, the results from PASTE show substantially worse performance (Figure S1d).

Supplementary Table

Technology	Tissue type	# slices	# samples
Spatial Transcriptomics	Breast cancer tumor	4	$\{254, 250, 263, 262\}$
Visium	Mouse cortex	2	$\{2805, 2874\}$
Slide-seqV2	Mouse hippocampus	2	$\{18651, 9247\}$

Table S1: **Spatial genomics datasets used in this paper.**

Supplementary Figures

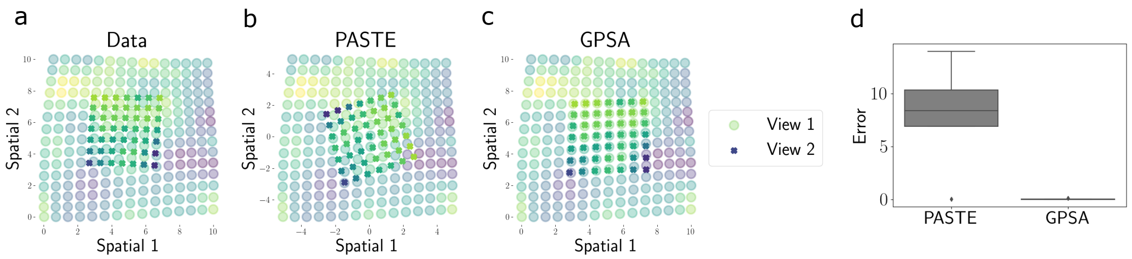

Figure S1: **Aligning synthetic samples with different fields-of-view.** (a) Synthetic data for two slices, one with a smaller field-of-view than the other. (b) Aligned coordinates from PASTE. (c) Aligned coordinates from GPSA. (d) Error for GPSA and PASTE aligned coordinates across five repetitions.

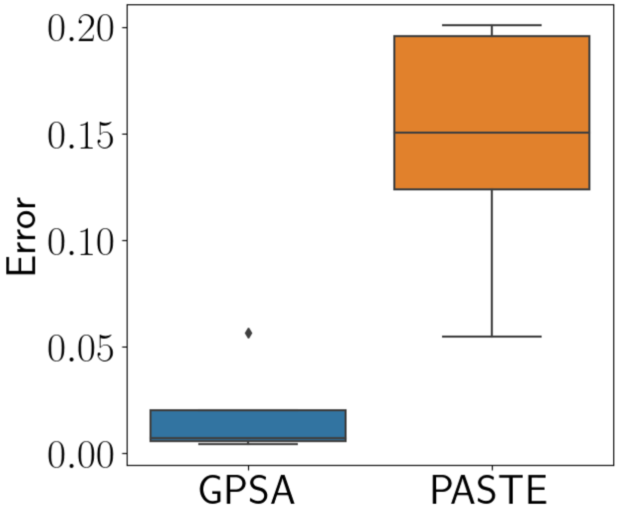

Figure S2: **GPSA scales to large number of spots.** Shown here is the alignment error for a synthetic dataset containing 2,500 spots. Error is computed for GPSA and PASTE alignments, with 10 repetitions. Each boxplot shows the minimum and the maximum values (whiskers), the median (center line), and the 1st and 3rd quartile (the box boundary); the outliers are plotted separately and removed from computation (here, for GPSA).

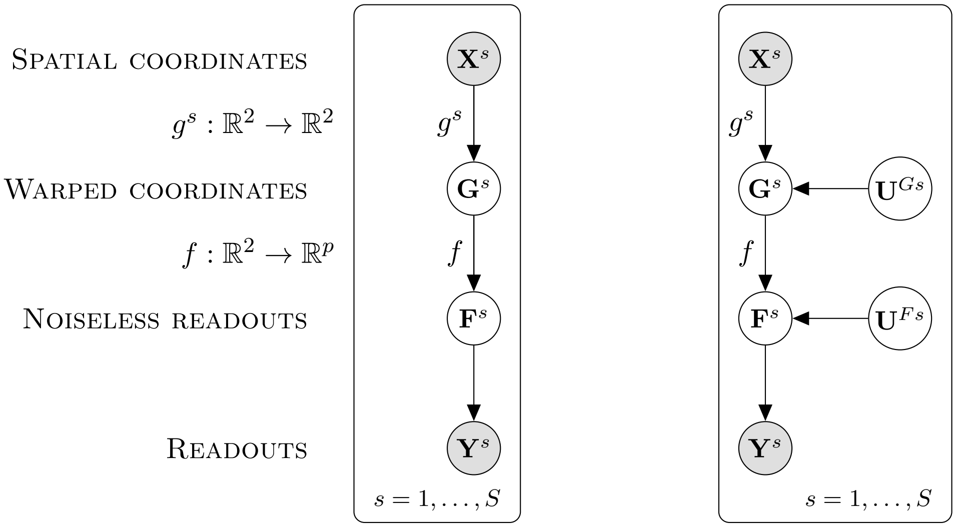

(a) **Graphical model for GPSA.** (b) **Graphical model for variational GPSA.**

Figure S3: **Graphical model representations for GPSA.** Each circle represents a random variable; shaded circles are observed random variables. The two Gaussian processes are found in the top two conditional distributions g^s and f. The conditional distribution for the readouts Y^s adds Gaussian noise to the noiseless readouts.

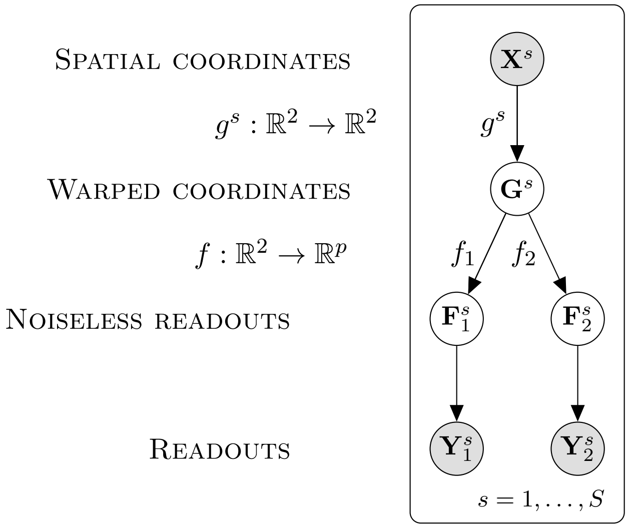

Figure S4: **Graphical model for multimodal GPSA.** As above, each circle represents a random variable; shaded circles are observed random variables. The two layers of Gaussian processes are found in the top two conditional distributions g^s and f_1, f_2. The conditional distribution for the readouts Y_*^s adds Gaussian noise to the noiseless readouts.

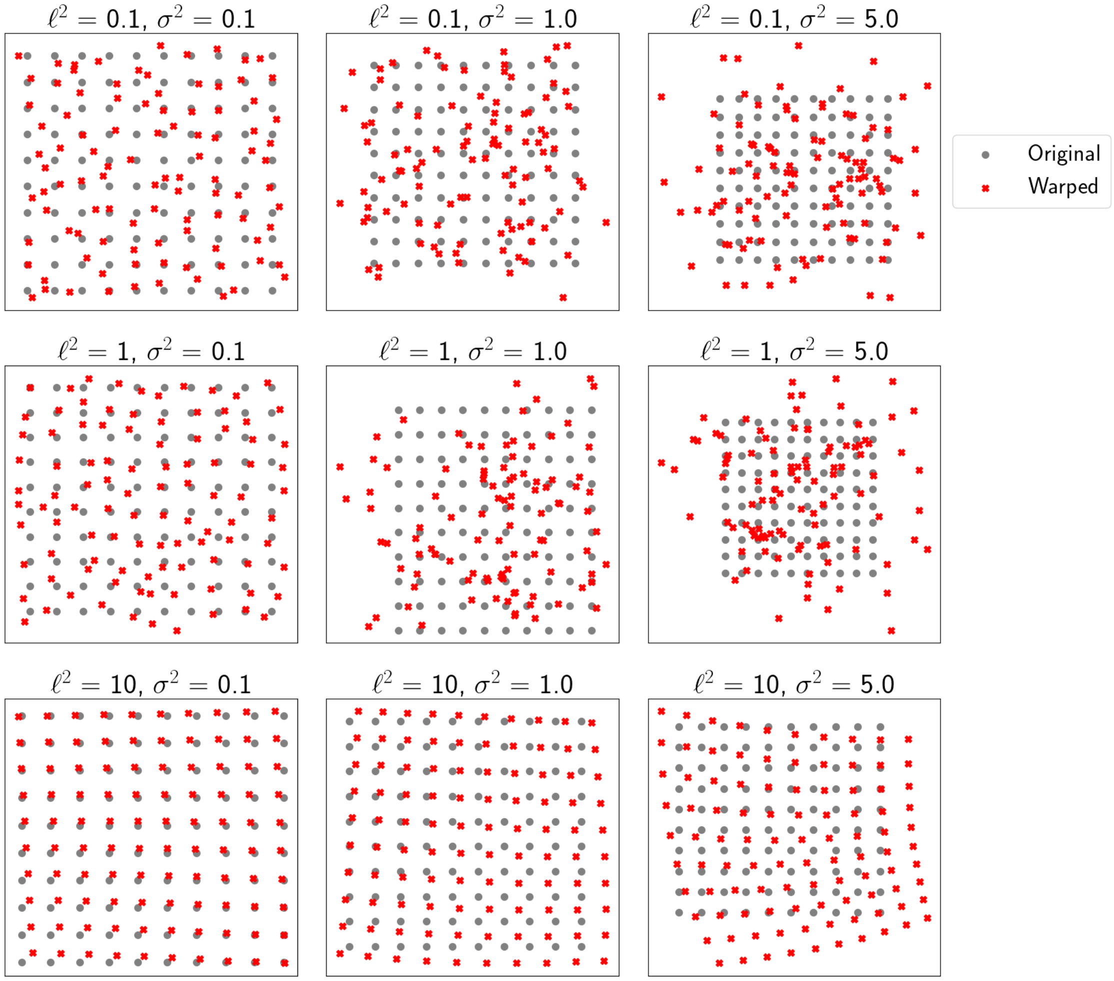

Figure S5: **Demonstration of spatial warp parameters.** Each panel shows a sampled warp for a setting of the lengthscale ℓ^2 and spatial variance σ^2 parameters. Gray points show the original unwarped spatial locations, and red points show the warped points after applying a warp sampled from the first layer of GPSA.

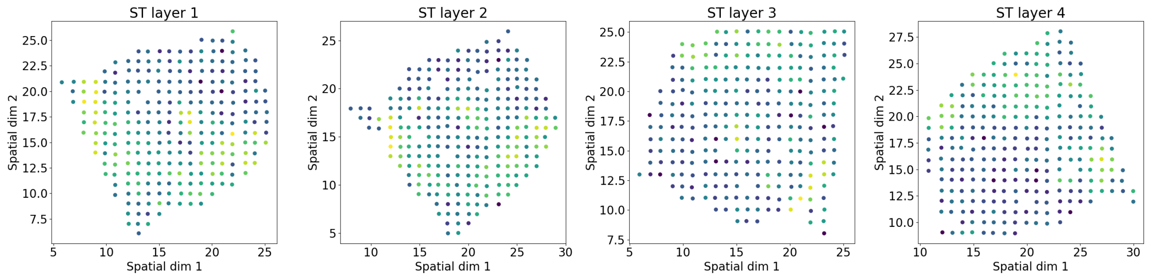

Figure S6: **Spatial Transcriptomics sample from breast cancer patient.** Each panel shows the spatial coordinates for one of the four slices, where each spot is colored by the log-expression of one gene at that spot.

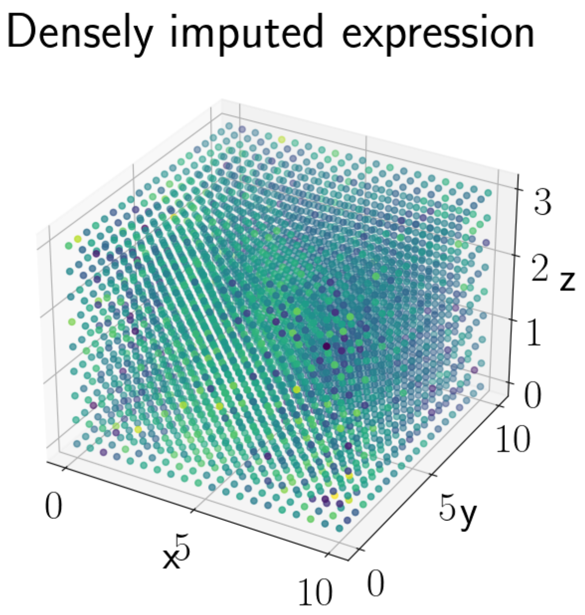

Figure S7: **Dense imputation of gene expression within the three-dimensional common coordinate system for the gene *FN1* in the ST breast cancer data.**

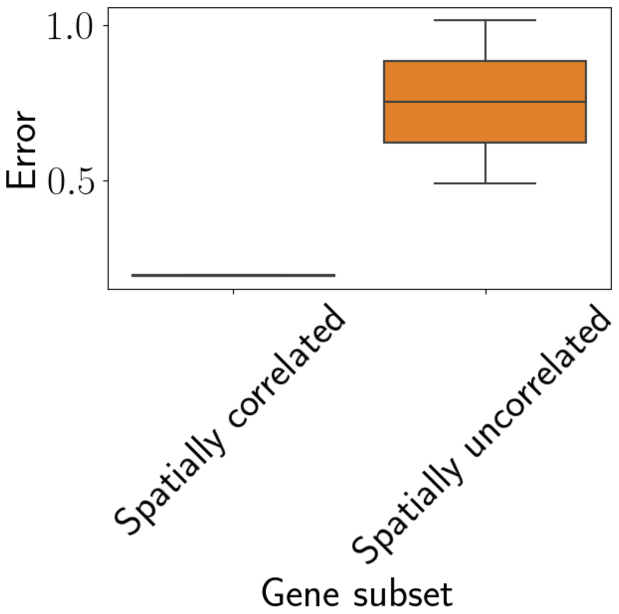

Figure S8: **Alignment error using spatially correlated and spatially uncorrelated genes on the breast cancer ST data.** We applied a synthetic GP-based warp to the ST data, fit GPSA, and computed the alignment error. The left box shows the results using five genes with the highest Moran's I score (spatially correlated genes), and the right box shows results using the five genes with the lowest Moran's I score (spatially uncorrelated genes). Each boxplot shows the minimum and the maximum values (whiskers), the median (center line), and the 1st and 3rd quartile (the box boundary).

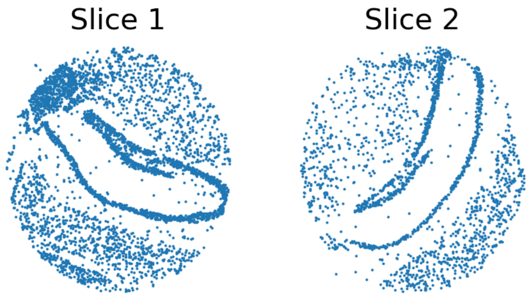

Figure S9: **Slide-seqV2 slices without initial coarse alignment.**

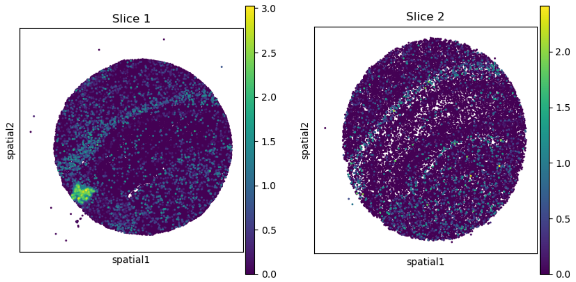

Figure S10: **Expression of the gene *ENPP2* in the two Slide-seqV2 slices.**

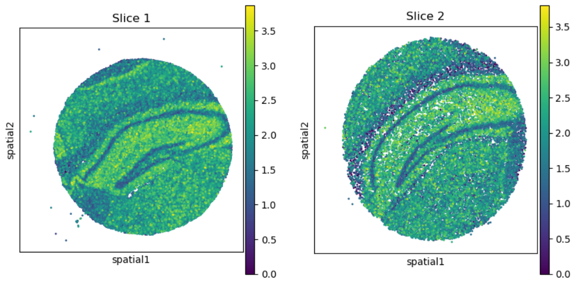

Figure S11: **Expression of the gene *MR-ND1* in the two Slide-seqV2 slices.**

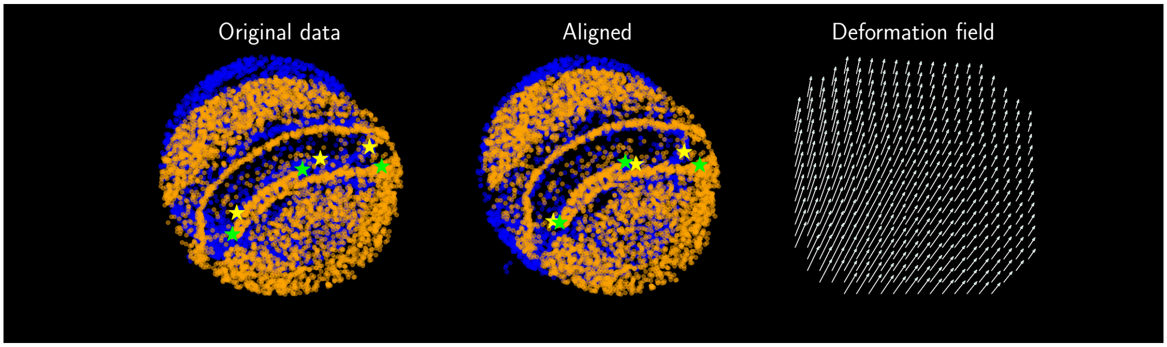

Figure S12: **Landmark locations in the Slide-seqV2 data.** Left: Manual annotations of three landmark locations in the original data for two slices. Spots for slice 1 and slice 2 are shown in blue and orange, respectively. Slice 1 landmarks are marked by yellow stars, and slice 2 landmarks are shown by green stars. Middle: Location of three landmarks after alignment with GPSA. Right: Deformation field showing the warping function learned by GPSA. Each arrow shows the direction of warping for a point in a grid across the second slice.

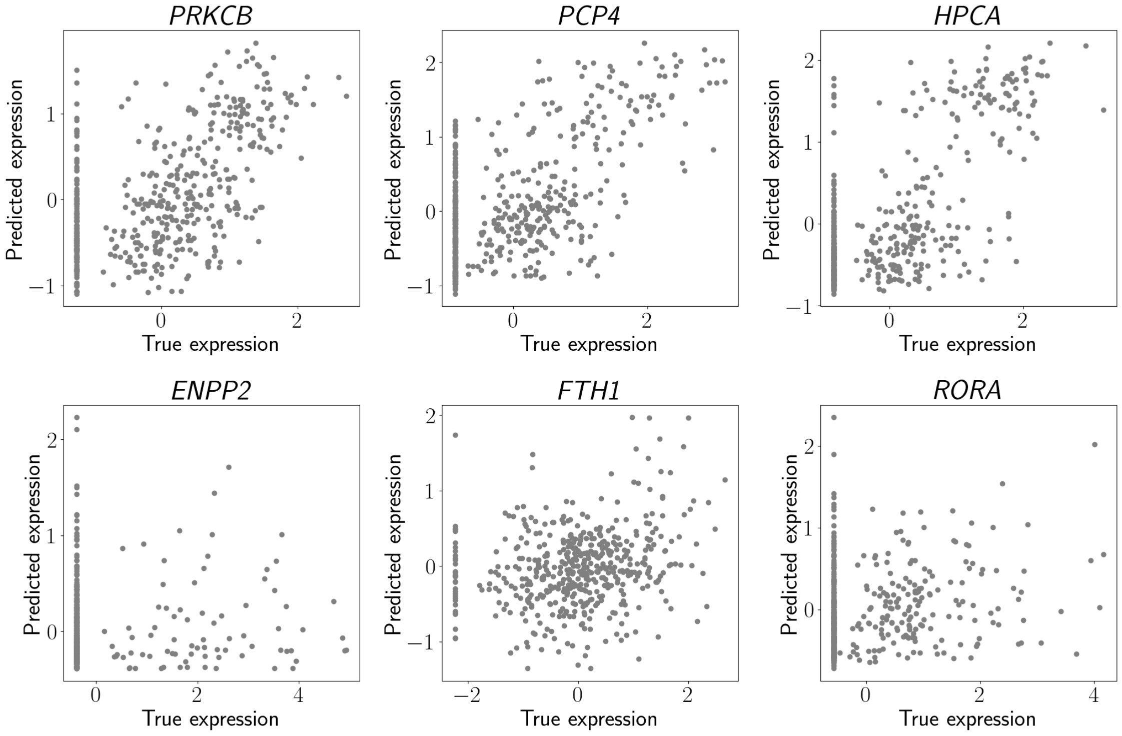

Figure S13: **Example predictions from prediction experiment with Slide-seqV2 data.** Shown are the true ($\log(x+1)$) and predicted values for six genes in the Slide-seqV2 prediction experiment. The top row shows the three genes for which the model had the best predictive performance, and the bottom row shows the three genes for which the model had the worst predictive performance.

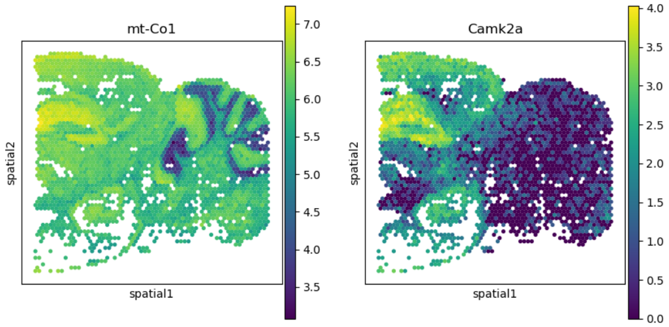

Figure S14: **Expression of *MT-CO1* and *CAMK2A* in the Visium mouse cortex.**

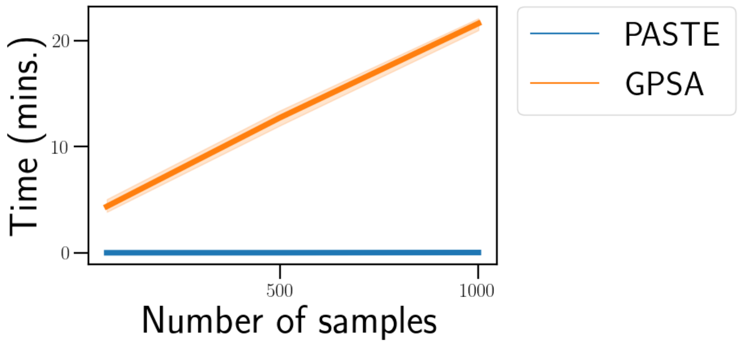

Figure S15: **Time complexity of GPSA and PASTE.**

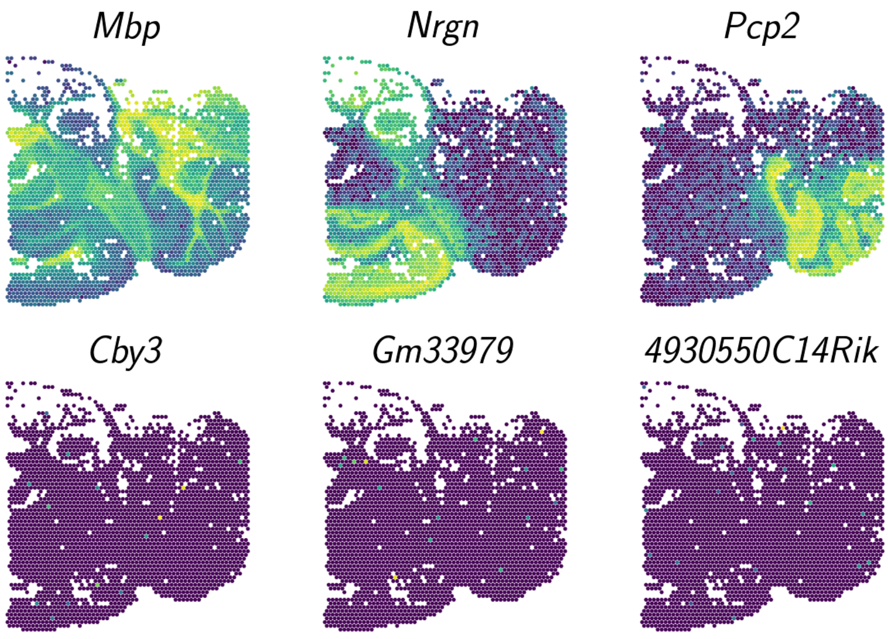

Figure S16: **Spatially variable and non-variable genes in the Visium mouse brain dataset.** The top row shows the a set of example genes that were spatially variable, and the bottom row shows an example set of genes that were not highly spatially variable.

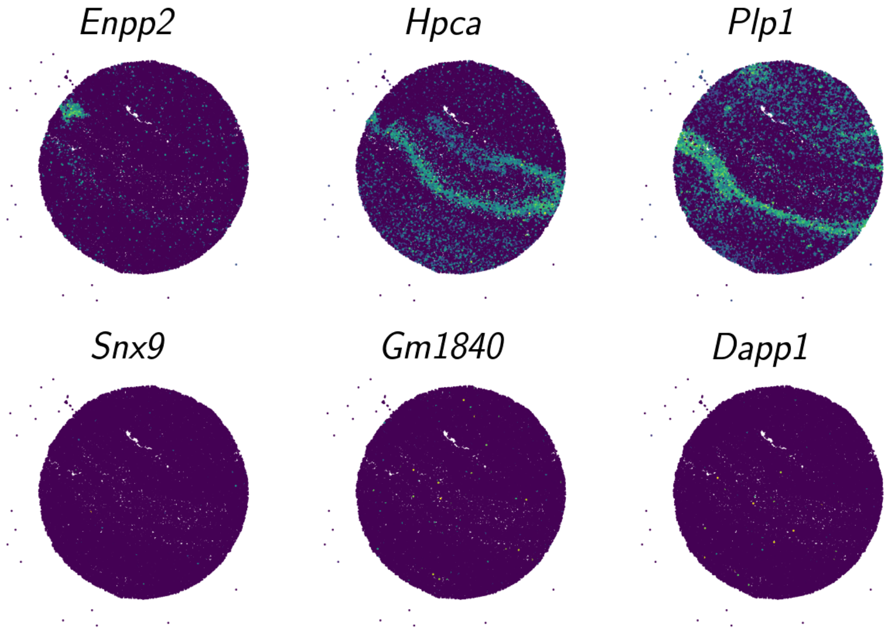

Figure S17: **Spatially variable and non-variable genes in the Slide-seqv2 mouse brain dataset.** The top row shows the a set of example genes that were spatially variable, and the bottom row shows an example set of genes that were not highly spatially variable.

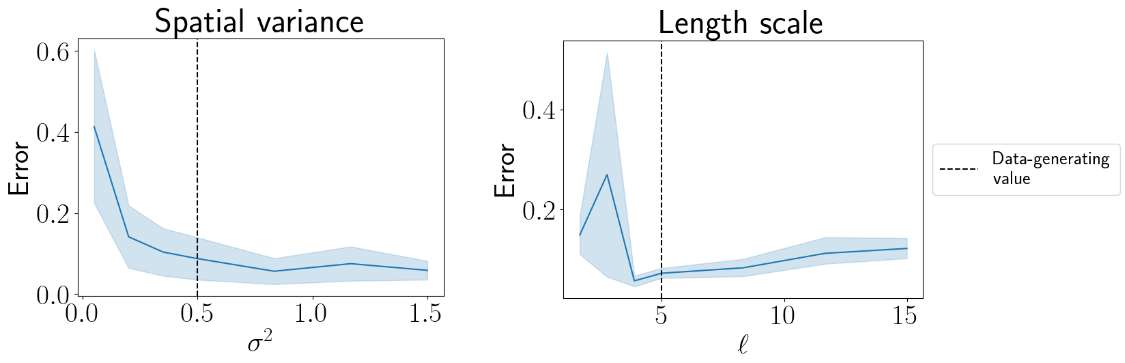

Figure S18: **Testing the robustness of GP covariance function parameters.** Left: MSE of GPSA alignment using synthetic data across a range of values for the fixed spatial variance parameter σ^2. Right: Same as left, expect for a range of values for the fixed length scale parameter ℓ.

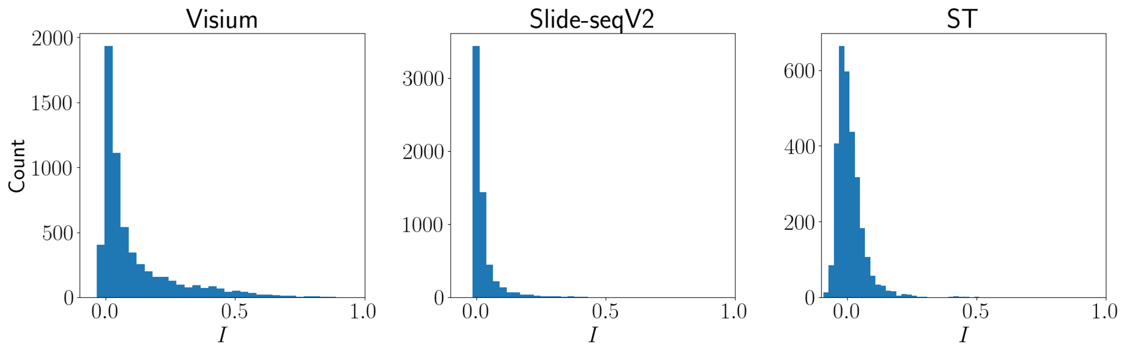

Figure S19: **Spatial autocorrelation in spatial genomics data.** Each panel shows a histogram of Moran's I statistic for each gene in the Visium, Slide-seqV2, and ST data.

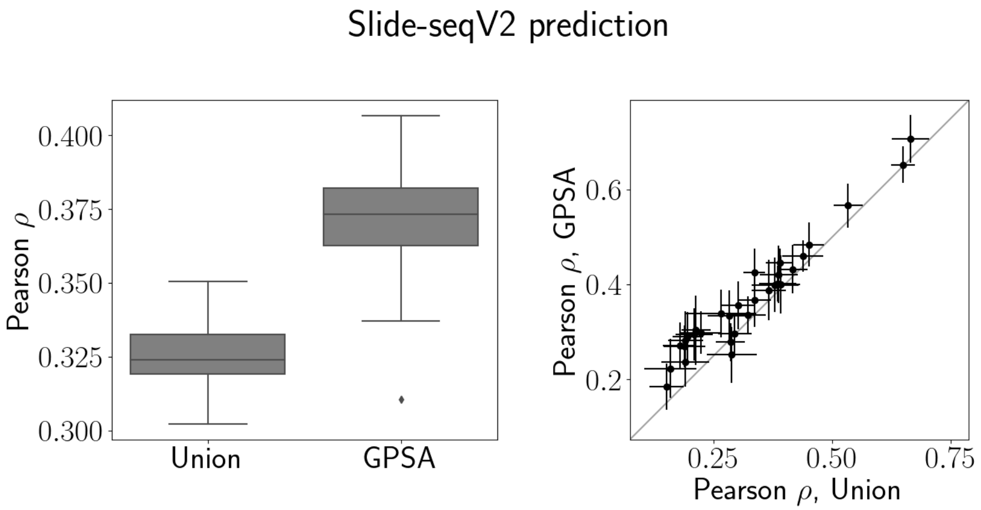

Figure S20: **Prediction experiment with Slide-seqV2 data.** Pearson correlation (ρ) for predictions of readout values versus ground truth on a held-out dataset. "Union" represents predictions from a GP fit to a naive concatenation of the observed samples, and "GPSA" refers to predictions from a GP fit jointly across all samples using the aligned coordinate system. Left: Mean ρ across genes for GPSA and Union approaches with 10 repetitions. Right: Prediction ρ for each gene, where points and error bars show the mean and standard deviation across repeated trials, respectively. Each boxplot shows the minimum and the maximum values (whiskers), the median (center line), and the 1st and 3rd quartile (the box boundary); the outliers are plotted separately and removed from computation (here, for GPSA).

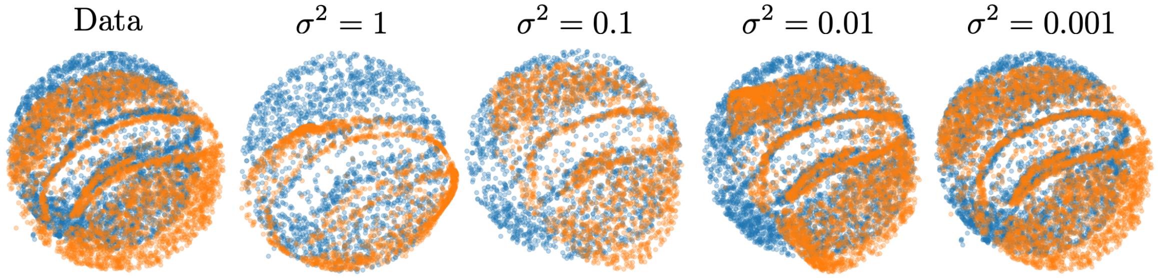

Figure S21: **Alignment of Slide-seqV2 data across range of regularization hyperparameter settings.** To regularize the model, we fixed the spatial variance hyperparameter σ^2 of the first-layer warping GP. Lower values of σ^2 restrict the warp from moving points far from their original locations, thus especially regularizing areas where there is little overlapping structure between the two slices. The left panel above shows the original spot locations, and the remaining panels show the aligned spot locations for decreasing σ^2.
