# Supplementary material for: Alignment of spatial genomics data using deep Gaussian processes
Source: Nat Methods. 2023 Aug 17;20(9):1379–87. doi: 10.1038/s41592-023-01972-2 (PMC10482692; doi:10.1038/s41592-023-01972-2)
Supplement: Supplementary file 2 — Reporting Summary [file 41592_2023_1972_MOESM2_ESM.pdf]

nature research

Corresponding author(s): Barbara Engelhardt

Last updated by author(s): Nov 17, 2022

Reporting Summary

Nature Research wishes to improve the reproducibility of the work that we publish. This form provides structure for consistency and transparency in reporting. For further information on Nature Research policies, see our Editorial Policies and the Editorial Policy Checklist.

Statistics

For all statistical analyses, confirm that the following items are present in the figure legend, table legend, main text, or Methods section.

n/a	Confirmed	
☐	☒	The exact sample size (n) for each experimental group/condition, given as a discrete number and unit of measurement
☐	☒	A statement on whether measurements were taken from distinct samples or whether the same sample was measured repeatedly
☐	☒	The statistical test(s) used AND whether they are one- or two-sided<br>*Only common tests should be described solely by name; describe more complex techniques in the Methods section.*
☐	☒	A description of all covariates tested
☐	☒	A description of any assumptions or corrections, such as tests of normality and adjustment for multiple comparisons
☐	☒	A full description of the statistical parameters including central tendency (e.g. means) or other basic estimates (e.g. regression coefficient) AND variation (e.g. standard deviation) or associated estimates of uncertainty (e.g. confidence intervals)
☐	☒	For null hypothesis testing, the test statistic (e.g. F, t, r) with confidence intervals, effect sizes, degrees of freedom and P value noted<br>*Give P values as exact values whenever suitable.*
☐	☒	For Bayesian analysis, information on the choice of priors and Markov chain Monte Carlo settings
☐	☒	For hierarchical and complex designs, identification of the appropriate level for tests and full reporting of outcomes
☐	☒	Estimates of effect sizes (e.g. Cohen's d, Pearson's r), indicating how they were calculated

Our web collection on statistics for biologists contains articles on many of the points above.

Software and code

Policy information about availability of computer code

Data collection	No software was used to collect the data.
Data analysis	Code for the model developed in this paper: https://github.com/andrewcharlesjones/spatial-alignment<br>Python scripts in the experiments/ directory of the above repository were used to run the experiments.<br>Other packages used:<br>numpy v1.19.2<br>pandas v1.3.5<br>scikit_learn v1.0.1<br>scipy v1.7.1<br>torch v1.8.0<br>gpsa v0.7

For manuscripts utilizing custom algorithms or software that are central to the research but not yet described in published literature, software must be made available to editors and reviewers. We strongly encourage code deposition in a community repository (e.g. GitHub). See the Nature Research guidelines for submitting code & software for further information.

Data

Policy information about availability of data

All manuscripts must include a data availability statement. This statement should provide the following information, where applicable:

- Accession codes, unique identifiers, or web links for publicly available datasets
- A list of figures that have associated raw data
- A description of any restrictions on data availability

ST data were obtained from the PASTE code repository: https://github.com/raphael-group/paste. All four layers from the sample_data/ directory were used.

Visium data were obtained from the 10x Genomics website. Data for the two slices were downloaded from the "Datasets" page. Specifically, spatial gene expression and H&E stains were downloaded from the following links:

- Mouse Brain Serial Section 1 (Sagittal-Posterior): https://www.10xgenomics.com/resources/datasets/mouse-brain-serial-section-1-sagittal-posterior-1-standard-1-1-0
- Mouse Brain Serial Section 2 (Sagittal-Posterior): https://www.10xgenomics.com/resources/datasets/mouse-brain-serial-section-2-sagittal-posterior-1-standard-1-1-0

Slide-seqV2 data were downloaded from the Broad Institute's Single Cell Portal: https://singlecell.broadinstitute.org/single_cell/study/SCP815/highly-sensitive-spatial-transcriptomics-at-near-cellular-resolution-with-slide-seqv2. Two pucks corresponding to the mouse hippocampus were used: Puck_191204_01 and Puck_200115_08.

Field-specific reporting

Please select the one below that is the best fit for your research. If you are not sure, read the appropriate sections before making your selection.

☒ Life sciences ☐ Behavioural & social sciences ☐ Ecological, evolutionary & environmental sciences

For a reference copy of the document with all sections, see nature.com/documents/nr-reporting-summary-flat.pdf

Life sciences study design

All studies must disclose on these points even when the disclosure is negative.

Sample size	All data used in this study is publicly available. Full sample sizes were used based on the size of the respective available public datasets.
Data exclusions	All data used in this study is publicly available. No data were excluded from these datasets.
Replication	For all computational experiments where repetitions were possible, we included multiple randomized repetitions of the experiments to ensure their replicability.
Randomization	All data used in this study is publicly available. We did not perform any randomization of the available data.
Blinding	All data used in this study is publicly available. We did not collect the data.

Reporting for specific materials, systems and methods

We require information from authors about some types of materials, experimental systems and methods used in many studies. Here, indicate whether each material, system or method listed is relevant to your study. If you are not sure if a list item applies to your research, read the appropriate section before selecting a response.

Materials & experimental systems

n/a	Involved in the study
☒	☐ Antibodies
☒	☐ Eukaryotic cell lines
☒	☐ Palaeontology and archaeology
☒	☐ Animals and other organisms
☒	☐ Human research participants
☒	☐ Clinical data
☒	☐ Dual use research of concern

Methods

n/a	Involved in the study
☒	☐ ChIP-seq
☒	☐ Flow cytometry
☒	☐ MRI-based neuroimaging
